# Differential Regulation of Genes Coding for Organelle and Cytosolic ClpATPases under Biotic and Abiotic Stresses in Wheat

**DOI:** 10.3389/fpls.2016.00929

**Published:** 2016-06-28

**Authors:** Senthilkumar K. Muthusamy, Monika Dalal, Viswanathan Chinnusamy, Kailash C. Bansal

**Affiliations:** ^1^ICAR-National Research Centre on Plant BiotechnologyNew Delhi, India; ^2^Division of Crop Improvement, ICAR-Indian Institute of Wheat and Barley ResearchKarnal, India; ^3^Division of Plant Physiology, ICAR-Indian Agricultural Research InstituteNew Delhi, India; ^4^ICAR-National Bureau of Plant Genetic ResourcesNew Delhi, India

**Keywords:** ClpATPases, *ClpB/HSP100*, *ClpC*, *ClpD*, *Triticum aestivum*, high temperature, salinity, oxidative stress

## Abstract

A sub-group of class I *Caseinolytic proteases* (*Clps*) function as molecular chaperone and confer thermotolerance to plants. We identified class I Clp family consisting of five *ClpB/HSP100*, two *ClpC*, and two *ClpD* genes from bread wheat. Phylogenetic analysis showed that these genes were highly conserved across grass genomes. Subcellular localization prediction revealed that TaClpC and TaClpD subgroup proteins and TaClpB1 proteins are potentially targeted to chloroplast, while TaClpB5 to mitochondria, and TaClpB2, TaClpB3, and TaClpB4 to cytoplasm. Spatio-temporal expression pattern analysis revealed that four *TaClpB* and *TaClpD2* genes are expressed in majority of all tissues and developmental stages of wheat. Real-time RT-PCR analysis of expression levels of *Clp* genes in seven wheat genotypes under different abiotic stresses revealed that genes coding for the cytosolic Clps namely *TaClpB2* and *TaClpB3* were upregulated under heat, salt and oxidative stress but were downregulated by cold stress in most genotypes. In contrast, genes coding for the chloroplastic Clps *TaClpC1, TaClpC2*, and *TaClpD1* genes were significantly upregulated by mainly by cold stress in most genotypes, while *TaClpD2* gene was upregulated >2 fold by salt stress in DBW16. The *TaClpB5* gene coding for mitochondrial Clp was upregulated in all genotypes under heat, salt and oxidative stresses. In addition, we found that biotic stresses also upregulated *TaClpB4* and *TaClpD1*. Among biotic stresses, *Tilletia caries* induced *TaClpB2, TaClpB3, TaClpC1*, and *TaClpD1*. Differential expression pattern under different abiotic and biotic stresses and predicted differential cellular localization of *Clps* suggest their non-redundant organelle and stress-specific roles. Our results also suggest the potential role of Clps in cold, salt and biotic stress responses in addition to the previously established role in thermotolerance of wheat.

## Introduction

*Caseinolytic protease* (*Clp*) was first identified in *Escherichia coli* as a heat shock inducible ATP-dependent protease complex that is capable of hydrolyzing casein (Hwang et al., 1987). Subsequent studies showed that ClpATPases fall within the AAA^+^ superfamily and it functions in assembly and disassembly of protein complexes (Neuwald et al., 1999; Frickey and Lupas, 2004). Members of this family form large hexameric ring structures in ATP dependent manner. These proteins have two AAA^+^ like domains, which consist of a RecA-like nucleotide-binding domain (NBD) and an α-helical domain (Lee et al., 2004). Under stress conditions, these proteases function as molecular chaperone by minimizing the protein aggregation and also target the damaged proteins for degradation (Snider et al., 2008). ClpATPases associate with ClpP, a proteolytic subunit for degradation of damaged proteins (Porankiewicz et al., 1999; Shikanai, 2001). This process maintains the quality of cellular proteins, thereby increases the tolerance of plants during stress conditions. ClpATPases are divided into two types namely; class I ClpATPases (ClpA, ClpB/Hsp100, ClpC, ClpD, and ClpE) and class II ClpATPases (ClpM, ClpN, ClpX, and ClpY). Class I ClpATPases have two ATP binding domains, whereas class II ClpATPases have one ATP binding domain (Singh et al., 2010). Class I ClpATPase (ClpB/HSP100, ClpC andClpD) gene families are well characterized in rice (*Oryza sativa*) and Arabidopsis (*Arabidopsis thaliana*) (Singh et al., 2010). Rice and Arabidopsis genomes encode 9 and 6 Class I ATPase family members, respectively (Singh et al., 2010). ClpB/HSP100 protein functions with the HSP70/DnaK chaperone system to remodel the denatured protein aggregates (Doyle and Wickner, 2009). In Arabidopsis and rice, the ClpB proteins have been divided into three groups based on sub-cellular localization namely, cytoplasmic ClpB (ClpB-cyt), chloroplastic ClpB (ClpB-p), and mitochondrial ClpB (ClpB-m) isoforms (Singh et al., 2010). Among three groups, the ClpB-Cyt/HSP100 is well studied in plants. It is involved in both basal and acquired thermotolerance, and also negatively influence primary root growth in maize (*Zea mays*) (Nieto-Sotelo et al., 2002). Arabidopsis ClpB-p is involved in chloroplast development (Myouga et al., 2006; Lee et al., 2007), while tomato (*Solanum lycopersicum*) ClpB-p (*LeHSP100/ClpB*) is necessary for acquired thermotolerance (Yang et al., 2006). *AtClpB-m* transcripts were upregulated during high temperature stress (Lee et al., 2007). In rice, *OsClpB-cyt, OsClpB-m*, and *OsClpB-p* expression were also upregulated by ABA treatment (Singh et al., 2010). Plant ClpC proteins function as stromal molecular chaperones involved in importing and protecting unfolded newly synthesized proteins, and play a vital role in chloroplast function, leaf development, and Fe homeostasis (Park and Rodermel, 2004; Sjögren et al., 2004; Adam et al., 2006; Wu et al., 2010). In Arabidopsis, *AtClpC* function as principle stromal protease responsible for maintaining homeostasis and also confers a novel protein quality control mechanism for chloroplast pre-protein import (Sjögren et al., 2014). In rice and Arabidopsis, *ClpD* expression was found to be induced by multiple abiotic stressessuch as dehydration, cold stress, high light, and during natural senescence (Kiyosue et al., 1993; Weaver, 1999; Zheng et al., 2002; Singh et al., 2010). Overexpression of *Arabidopsis AtHSP101* conferred the basal thermotolerance in transgenic rice (Katiyar-Agarwal et al., 2003). Thus, class I Clp proteins play an important role in stress responses, growth and development of plants.

Wheat is an important staple food and its production needs to be increased upto 50% to meet the food demand of the growing population by the end of 2050 (Godfray et al., 2010). One of the limitations to achieve this target is abiotic stresses that drastically reduce the wheat grain production (Viswanathan and Khanna-Chopra, 2001). Among abiotic stresses, high temperature stress adversely impacts wheat grain production in tropical and sub-tropical areas of the world (Ortiz et al., 2008; Lobell et al., 2012). An increase in temperature by 1°C above optimum during reproductive stage may reduce the wheat yield by 4–10% (Aggarwal et al., 2000; Brisson et al., 2010). Despite its importance, the genetic basis of heat tolerance is poorly understood, and no major QTLs (Quantitative Trait Loci) have been identified in wheat. At cellular level, heat shock proteins, also known as molecular chaperons, are the most studied candidate genes for high temperature response. Since class I Clp proteins perform important role in stress responses in different plant species, we examined the potential role of this class of genes in different stresses including high temperature stress in wheat. This study was conducted to determine the structural aspects and expression pattern of class I ClpATPases in wheat. The results revealed differential expression pattern of wheat class I ClpATPases in different tissues and developmental stages and in response to different stresses, and thus suggest their potential roles in biotic and abiotic stress tolerance of wheat.

## Materials and methods

### Identification and classification of Class I *ClpATPases* genes in wheat

The protein sequences of Class I *ClpATPase* genes from Arabidopsis (3 ClpB proteins—At1g74310, At2g25140, At5g15450; 2 ClpC proteins—At3g48870, At5g50920; and 1 ClpD protein—At5g51070) (Zheng et al., 2002) and rice (3 ClpB proteins—Os05g44340, Os02g08490, Os03g31300; 4 ClpC proteins—Os04g32560, Os12g12580, Os11g16590, Os1g16770; and 2 ClpD proteins—Os02g32520, Os04g33210) (Singh et al., 2010) were used as query and blastp search was performed in TriFLDB (http://TriFLDB.psc.riken.jp/) (Mochida et al., 2009) and NCBI database to identify the wheat class I *ClpATPase* gene homologs. Clustal omega (http://www.ebi.ac.uk/Tools/msa/clustalo/) and MEGA 5 software (Tamura et al., 2011) were used for removal of redundant cDNA clones. The identified wheat Class I ClpATPase homologs were reconfirmed by tblastn search in rice genome database TIGR (Ouyang et al., 2007) and in Arabidopsis genome database TAIR (https://www.arabidopsis.org/). Isoelectric point (pI) and molecular weight (Mw) was computed through ExPASy tool (http://web.expasy.org/compute_pi/). Protein subcellular localization was predicted through WoLF PSORT tool (Horton et al., 2007) and TargetP 1.1 server (Emanuelsson et al., 2007).

### Multiple sequence alignment, phylogenetic analysis, and domain prediction

Multiple sequence alignment was carried out using Clustal omega (http://www.ebi.ac.uk/Tools/msa/clustalo/) and Simple Modular Architecture Research Tool (SMART), (Letunic et al., 2012) was used to predict the conserved domains in class I ClpATPase protein sequences. The COILS Server (http://www.ch.embnet.org/software/COILS_form.html) was used to predict the coiled-coil region in TaClp proteins (Lupas et al., 1991). Rice, class I ClpATPase protein sequences were used as query and tblastn search was performed in phytozome database (https://phytozome.jgi.doe.gov/pz/portal.html) against the available grass genomes such as maize (*Z. mays*), sorghum (*Sorghum bicolor*), brachypodium (*Brachypodium distachyon*) and setaria (*Setaria italica*) to identify the respective homologs. For phylogenetic analysis, the amino acid sequences of Clp proteins from Arabidopsis and grass genomes were aligned using MUSCLE program (Tamura et al., 2011). Phylogenetic trees were constructed using Neighbor Joining method with pairwise deletion and poisson correction in MEGA 5 software (http://megasoftware.net/) (Tamura et al., 2011). Bootstrap analysis was performed with 2000 replicates to evaluate the reliability of different phylogenetic groups. MEME Suite web server was used to identify the conserved motifs in class I ClpATPase protein sequences (Bailey et al., 2009). Exon/Intron organization of class I *ClpATPase* genes were generated using gene structure display server program (Hu et al., 2015).

### Digital expression analysis

A blastn search was carried out using the identified *TaClps* genes against *Triticum aestivum* NCBI UniGene database to identify their UniGene IDs. The EST sequences of wheat *Clp* genes corresponding to their UniGene IDs were downloaded from NCBI UniGene database for digital expression analysis. These ESTs expression information were used to characterize the tissue specific expression of *TaClp* genes (**Table 3**). We also used wheat array expression data information to study the spatio-temporal expression pattern, abiotic and biotic stress inducible expression pattern of identified wheat *Clp* genes (except for *TaClpC2*, which doesn't have probe in affymetrix wheat genome array). Genevestigator v3 database was used for normalization and meta-analysis of the wheat array expression datasets (https://genevestigator.com/gv/doc/intro_plant.jsp) (Hruz et al., 2008). The following abiotic and biotic wheat array experiment's datasets were used in our study: NCBI GEO Accession GSE12936 (Chain et al., 2009), GSE21386 (Wang et al., 2010), GSE22080 (unpublished), GSE34445 (Zhu et al., 2012), GSE9915 (Bolton et al., 2008), GSE13660 (Desmond et al., 2008), GSE31762 (Krugman et al., 2010), and EBI Array Express IDs E-MEXP-1193 (Wan et al., 2008), E-MEXP-971 (Mott and Wang, 2007), E-MEXP-1488 (Ergen et al., 2009), and E-MEXP-1523 (Qin et al., 2008). The details of the wheat array experiments used in study were given in Supplementary Table S2.

### Plant materials and growth conditions

Seven genotypes of wheat (*T. aestivium*) cultivars WR544, C306, DBW16, NIAW34, PBW343, HD2687, and HW2019 were used in this study. The cultivars WR544, C306, DBW16, and NIAW34 were thermotolerant, whereas PBW343, HD2687, and HW2019 were thermosensitive. Seeds of these genotypes were germinated on cotton bed in falcon tubes as described by Singh et al. (2010). Seedlings were grown at 22–25°C under 16 h/8 h light/dark regime, respectively, in the culture room. Plants were watered regularly at an interval of 2–3 days to retain optimum moisture for plant growth. Fifteen days old seedlings with uniform size were selected and used for stress treatments and molecular analysis.

### Stress treatments

High temperature stress was imposed to the plants by placing the plants growing in the falcon tube in an incubator at 42 (±2)°C for 3 h. Cold stress was imposed by placing the plants in cold chamber at 2 (±2)°C for 3 h. Salt stress was imposed by saturating the cotton pad with 150 mM NaCl and sampling was done after 6 h. For oxidative stress, seedlings were treated with 10 mM H_2_O_2_ and kept in dark conditions for 6 h. Well watered seedlings grown at 22–25°C under 16 h/8 h light/dark regime in the culture room served as control. After the treatments, leaf tissues from 10 plants were pooled and immediately frozen in liquid nitrogen and stored at −80°C till further use.

### qRT-PCR analysis

High quality RNA was extracted using RNeasy® plant mini kit (QIAGEN) by following the manufacturer's instructions. Further the extracted RNA was subjected to DNaseI (Fermentas) treatment to obtain DNA-free RNA. First strand cDNA was synthesized from 1 μg of total RNA by using Superscript–III reverse transcriptase (Invitrogen, USA). Three biological replicates were used for qRT-PCR (We isolated RNA from three different samples from the pooled leaf tissues of each treatment of each genotypes). For qRT-PCR analysis, the cDNA was diluted to 1:4 times and 1.5 μL of diluted cDNA was used as a template in a 10 μL reaction volume in 96 well plates by following the manufacturer's instruction. List of primers used in this study is given in Supplementary Table S3. Quantitative RT-PCR analysis was carried out by using the Realplex^4^ system (Eppendorf) using iQ™ SYBR® Green (Bio-Rad). Expression data was normalized using endogenous control ubiquitin [Unigene ID Ta50503] gene expression. The expression was represented in the form of relative fold change which is the relative change in expression of *TaClp* genes under stress conditions as compared with their expression under control conditions. Expression level of *TaClp* gene in control condition was considered as 1. Relative fold change was calculated using the 2^−ΔΔCt^ method (Livak and Schmittgen, 2001). The error bars represents the standard deviation of the expression of the three biological replicates. A two-tailed unpaired *t*-test was performed to analyze the significance of the difference of the gene expression between control and stress condition.

## Results

### Identification and classification of class I *Clp* genes in wheat

**Figure 1:**
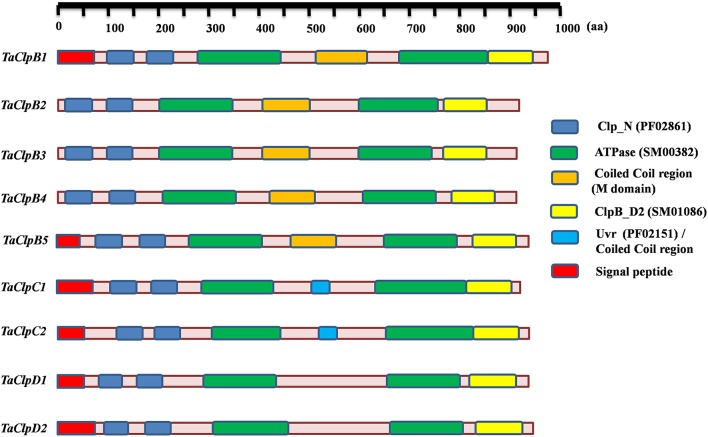
**Diagrammatic illustration of conserved domains position present in wheat ***TaClp*** genes**. The conserved domains were predicted using SMART tool.

Arabidopsis genome encodes 6 Class I ClpATPases, while rice genome encodes 9 Class I ClpATPases (Zheng et al., 2002; Singh et al., 2010). The protein sequences of Class I ClpATPases from Arabidopsis and rice were used as query for blastp search in the wheat database, TriFLDB (Mochida et al., 2009) and NCBI database to identify the respective wheat homologs. Initially 16 wheat cDNA clones were identified. Seven cDNA sequences were redundant and hence eliminated based on multiple sequence alignment analysis. Wheat ClpATPase homologs were reconfirmed by performing tblastn search in rice genome annotation project (RGAP) database and Arabidopsis genome (TAIR) database. Finally, nine wheat Class I ClpATPase homologs were identified which include five *ClpB* genes, two *ClpC* genes, and two *ClpD* genes (Table 1). The subcellular localization of wheat Clp proteins were predicted through WoLF PSORT tool (Horton et al., 2007) and TargetP 1.1 server (Emanuelsson et al., 2007). *TaClpB2, TaClpB3*, and *TaClpB4* localized to cytoplasm, *TaClpB5* localized to mitochondria, while *TaClpB1, TaClpC1, TaClpC2, TaClpD1*, and *TaClpD2* were localized to chloroplast (Table 1). Presence of a plastid transit peptide in the N-terminal of TaClpB1, TaClpC1, TaClpC2, TaClpD1, and TaClpD2 proteins, and a mitochondrial targeting sequence in the N-terminal of TaClpB5 conformed to the subcellular localization prediction. Simple Modular Architectural Related Tool (SMART) (Letunic et al., 2012) was used to predict and locate the conserved functional domains present in wheat Class I ClpATPases proteins (Table 2). The position of the conserved domains of the wheat Clp proteins were pictorially represented in Figure 1. All the wheat Clp proteins have two conserved Clp_N domains (PF02861) in the N-terminal and two ATPase domains which are essential for hexamerization and chaperone function (Krzewska et al., 2001; Weibezahn et al., 2004). A coiled coiled domain (M domain) located between the two nucleotide binding domains (NBD) is characteristic of ClpB proteins (Mishra and Grover, 2015). The M domain is variable in both sequence and length. It is absent in ClpA, while presence of a shorter M domain is predicted for ClpC and ClpD proteins in higher plants (Mishra and Grover, 2015). Our results revealed that M domain is present in ClpB and ClpC proteins while it is absent in ClpD proteins of wheat (Figure 1). The coiled coil region (M Domain) of ClpC1 (510–535) and ClpC2 (530–550) is located within the Uvr domain (PF02151), and is shorter in ClpC than that of ClpB (Figure 1 and Table 2). M domain can adapt different orientation in a single hexamer. The horizontal orientation represses the ClpB activity by preventing binding of Hsp70, while tilted orientation activates the ClpB activity (Carroni et al., 2014). The interaction of M domain of ClpB with Hsp70 chaperone system is essential for regulating the intrinsic disaggregase activity of ClpB hexamer (DeSantis and Shorter, 2012; Carroni et al., 2014). Similar to wheat ClpC, the M domain of bacterial ClpC is half the size of that in ClpB and it corresponds to the N terminal coiled coil (M domain) region of ClpB (Mishra and Grover, 2015; Nishimura and van Wijk, 2015). The N-domain and the NBD1-M domain of bacterial ClpC interacts with the C-terminal domain of MecA which facilitates hexamerization and activation of ClpC. This ClpC-MecA complex plays an important role in disaggregation and translocation of the unfolded proteins to ClpP for degradation (Schlothauer et al., 2003; Kirstein et al., 2006; Wang et al., 2011).

**Table 1:** **Wheat ***ClpB/Hsp100, ClpC***, and ***ClpD*** genes and their predicted cellular localization**.

**Gene**	**TriFLDBcDNA sequence id**	**NCBI GenBank ID**	**Size (aa)**	**Mw (K Da)**	**Pi**	**Cellular localization**
*TaClpB1*	tplb0010c03	AK330787	975	108.73	6.66	Chloroplast
*TaClpB2*	RFL_Contig4216	AK333710	918	101.06	5.84	Cytoplasm
*TaClpB3*	AF174433	AF174433	913	100.90	5.95	Cytoplasm
*TaClpB4*	RFL_Contig5540	AK335181	913	99.79	6.12	Cytoplasm
*TaClpB5*		JV864025.1	933	104.12	5.54	Mitochondria
*TaClpC1*	RFL_Contig3528	AK332945	920	101.80	6.55	Chloroplast
*TaClpC2*	RFL_Contig4439	AK333957	938	102.74	6.26	Chloroplast
*TaClpD1*	RFL_Contig1508	AK330701	937	101.78	6.39	Chloroplast
*TaClpD2*	RFL_Contig3864	AK333318	946	102.67	8.74	Chloroplast

*aa, amino acids; pI, isoelectric point; Mw, Molecular weight. The subcellular localization of Clps were predicted through WoLF PSORT tool and TargetP 1.1 server*.

**Table 2:** **Functional domains identified in wheat ClpB, ClpC, and ClpD proteins**.

**Gene**	**Signal peptide length**	**Clp_N (PF02861)**	**ATPase domain**	**Coiled coil domain (M domain)**	**ClpB_D2 small domain**	**Uvr domain**
*TaClpB1*	71	97–149, 174–225	278–442, 681–856	518–613	855–949	
*TaClpB2*	–	17–69, 98–148	202–347, 600–759	407–503	770–860	
*TaClpB3*	–	17–69, 98–148	201–346, 599–742	406–502	768–859	
*TaClpB4*	–	17–66, 104–154	209–354, 609–752	421–511	780–871	
*TaClpB5*	40	52–104, 129–180	233–378, 636–778	446–568	811–900	
*TaClpC1*	70	104–156, 179–231	290–430, 633–814	510–535	813–904	507–542
*TaClpC2*	51	119–171, 194–246	307–447, 651–832	530–550	831–931	524–559
*TaClpD1*	43	81–127, 157–208	292–440, 640–820		829–919	
*TaClpD2*	89	94–144, 172–224	305–454, 659–806		839–929	

*SMART tool was used to predict and locate the position of conserved domains and COILS Server was used to predict Coiled coil domain (M domain)*.

### Phylogenetic relationship of class I clpatpases of family

**Figure 2:**
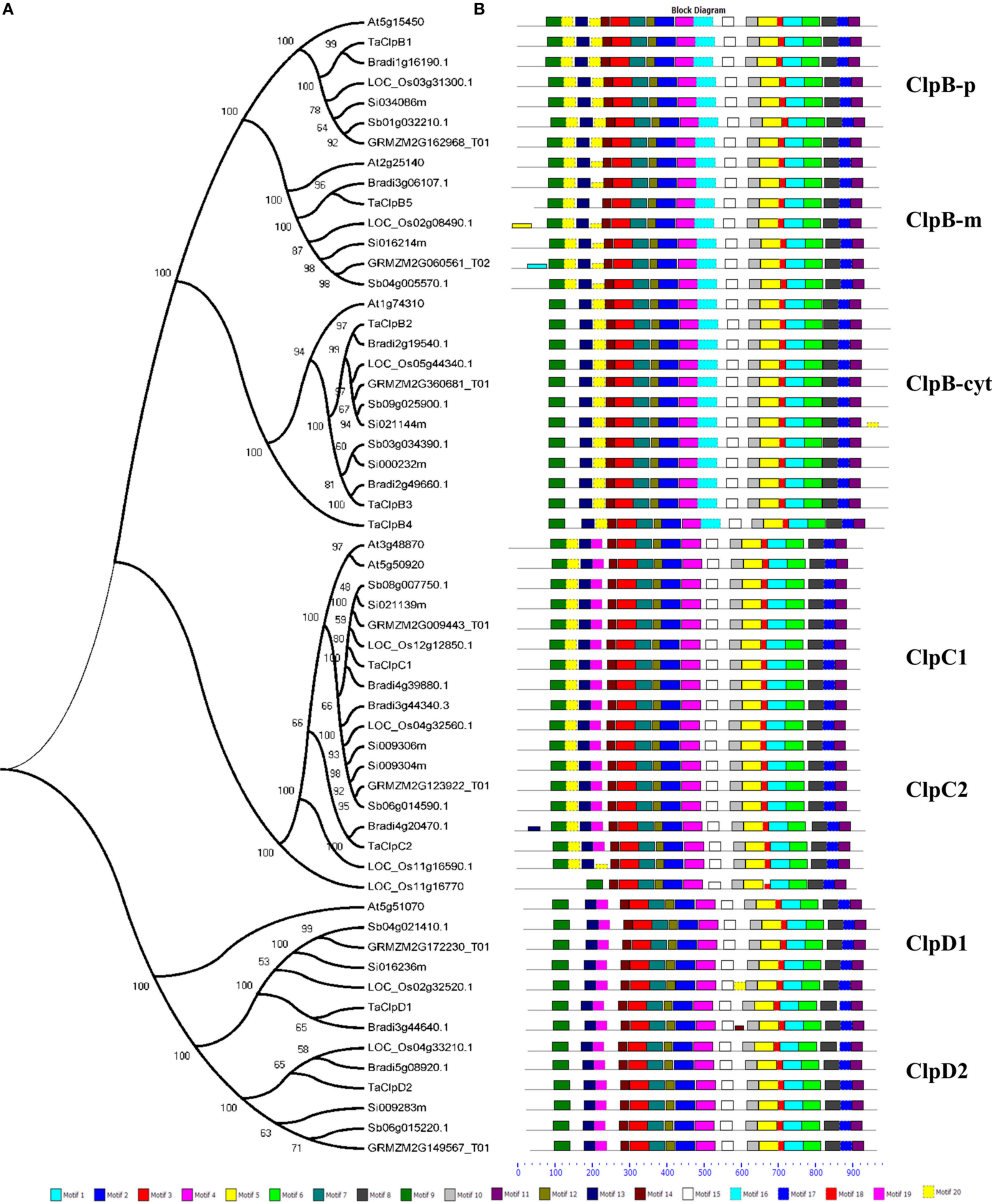
**Phylogenetic relationship and conserved motif compositions of wheat Clp proteins and their respective grass homologs. (A)** Multiple sequence alignment of 57 full-length grass Clp proteins was done using MUSCLE and phylogenetic tree was constructed using MEGA5. The number at each node represents the bootstrap value from 2000 replicate. Protein IDs—At, *Arabidopsis thaliana*; LOC_Os, *Oryza sativa*; Sb, *Sorghum bicolor*; GRMZM, *Zea mays*; Bradi, *Brachypodium distachyon;* Si, *Setaria italica*. **(B)** Schematic representation of the conserved motifs in the Clp proteins as revealed by MEME analysis. Each motif is represented by a box numbered at the bottom.

Blastp search was performed using rice Class I ClpATPases proteins against publicly available grass genomes (maize, sorghum, brachypodium, and setaria) in Phytozome database and the respective grass Clp protein homologs were downloaded. Our analysis led to the identification of seven Clp proteins from maize, eight Clp proteins from sorghum and nine Clp proteins each from brachypodium and setaria (Supplementary Table S1). Maize encode for 3, 2, and 2 *ClpB, ClpC*, and *ClpD* genes, respectively, sorghum encode for 4, 2, and 2 *ClpB, ClpC*, and *ClpD* genes, respectively, while brachypodium and setaria encode 4, 3, and 2 *ClpB, ClpC*, and *ClpD* genes, respectively (Supplementary Table S1). The multiple sequence alignment of wheat Clp proteins with their respective grass Clp protein homologs revealed that the proteins TaClpB1, TaClpB2, TaClpB3, and TaClpC1 were highly conserved across the grass genomes (Supplementary Figures 1A–C). We also studied the phylogenetic relationship of wheat class I ClpATPases proteins with their respective grass Clp protein homologs and Arabidopsis homologs using MEGA 5 software (Tamura et al., 2011). The analysis showed that Clp proteins from grass genomes and Arabidopsis genome falls into three groups designated as ClpB, ClpC, and ClpD (Figure 2), in accordance with rice and Arabidopsis Clp proteins classification (Singh et al., 2010). Within each subclass, Clp proteins from grass genome clustered together suggesting that they were highly conserved across grass genomes (Figure 2).

**Figure 3:**
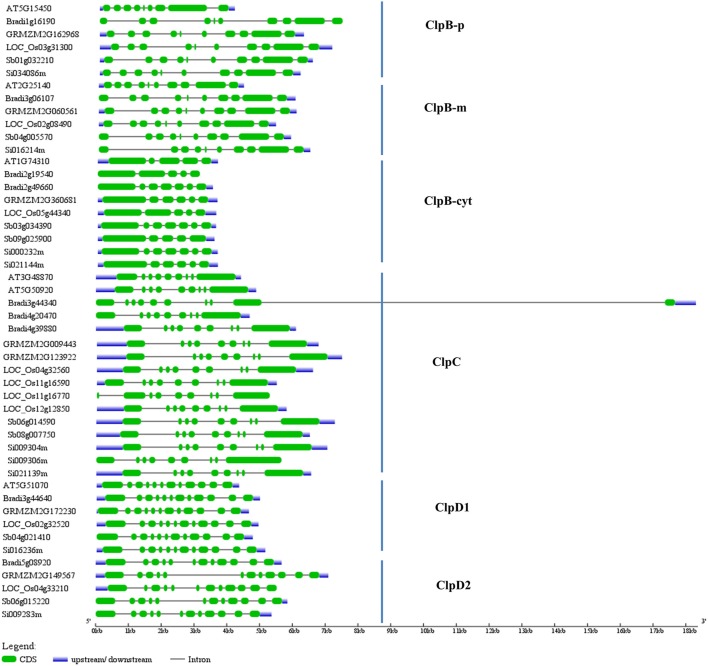
**Exon/intron organization of grass ***Clp*** genes**. The blue lines represent 5′-UTR or 3′-UTR, green boxes represent exons and black lines indicate introns. The Clp genomic sequences and CDS sequences were downloaded from Phytozome and these sequences were compared in gene structure display server program to infer the exon/intron organization of grass *Clp* genes. Gene IDs—At, *Arabidopsis thaliana*; LOC_Os, *Oryza sativa*; Sb, *Sorghum bicolor*; GRMZM, *Zea mays*; Bradi, *Brachypodium distachyon;* Si, *Setaria italica*.

We also studied the exon/intron organization of Arabidopsis and grass *Clp* genes. For these we downloaded the grass *Clp* genomic and CDS sequences from Phytozome database and these sequences were compared in gene structure display server program to infer the exon/intron organization. Interestingly *ClpB-m, ClpC1, ClpD1*, and *ClpD2* family genes of Arabidopsis and grass genomes displayed similar exon-intron structures (Figure 3). *ClpB-m* family genes contain 10 exons, and grass *ClpD1* and *ClpD2* family genes contain 12 exons each (Figure 3). Arabidopsis *ClpB-p* has 9 exons, whereas grass *ClpB-p* family genes contain 10 exons (Figure 3).

### Expression analysis of class I *Clps*

#### Digital expression analysis

**Figure 4:**
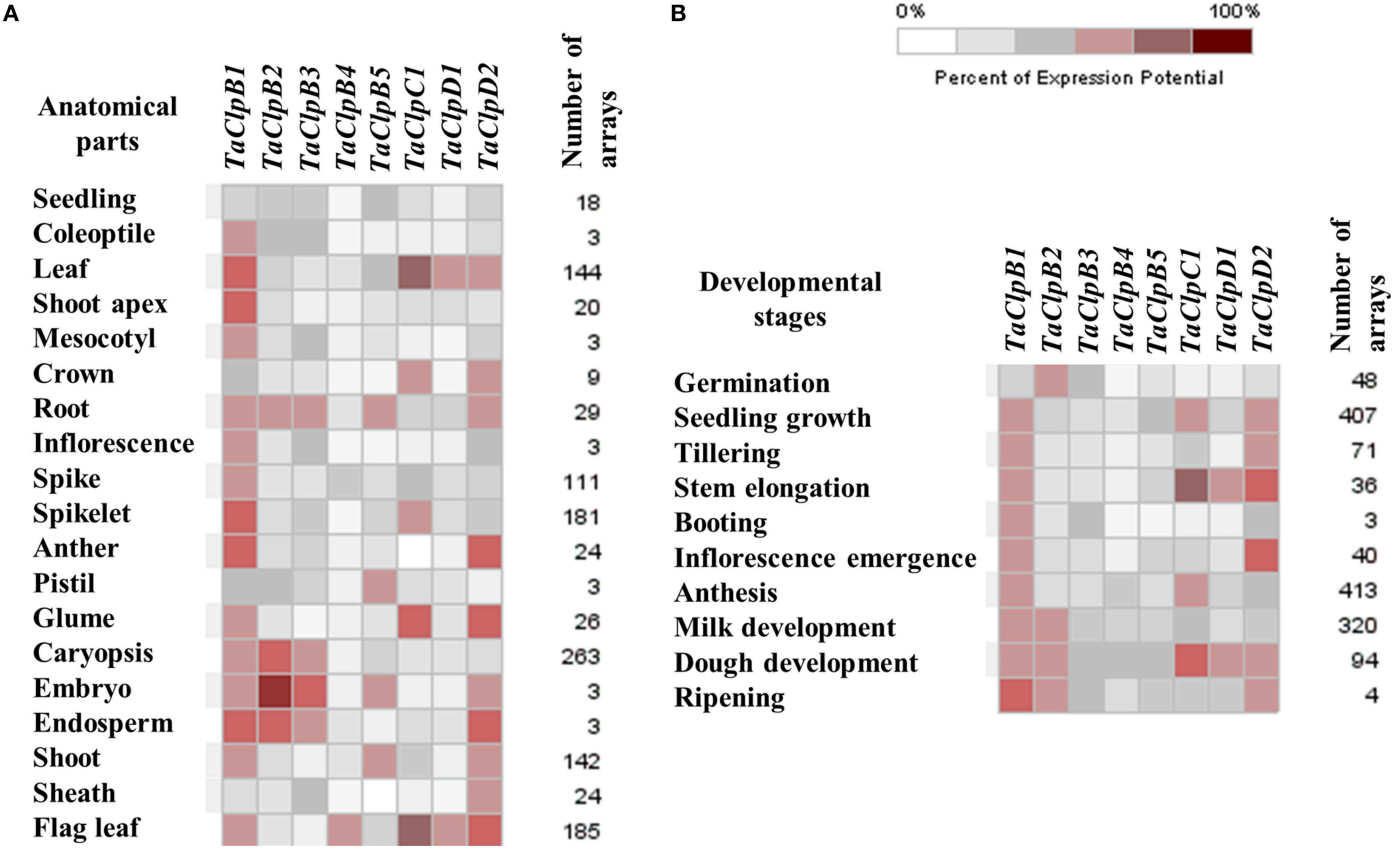
**Array based expression profiles of ***TaClp*** genes in various tissues and developmental stages of wheat. (A)** Anatomical tissues and **(B)** Developmental stages. The expression levels of *TaClp* genes are presented as heat maps generated using meta-analysis tool at Genevestigator (V3) http://www.genevestigator.ethz.ch. These expression analyses were performed using normalized wheat genome array expression datasets.

UniGene ID information of wheat *Clp* genes was retrieved from NCBI database (Table 3). These UniGene IDs were used to retrieve the respective *Clp* gene EST sequences along with their respective cDNA library information from NCBI UniGene database. Based on this, expression of these genes were assigned into five tissues namely leaf, root, stem, inflorescence, and developing seeds (Table 3). We also studied the spatio-temporal expression pattern, abiotic, and biotic stress inducible expression pattern of identified wheat *Clp* genes (except for *TaClpC2*, for which the probe was not available in affymetrix wheat genome array) using wheat genome array expression datasets through Genevestigator v3 database (Hruz et al., 2008). We found that all the nine wheat *Clp* genes were supported by expression data (Table 3, Figures 4A,B). All the *TaClp* genes except *TaClpB4* expressed in most of the anatomical tissues and in all 10 developmental stages (Figures 4A,B). However, there were significant differences in the level of expression of these genes. The expression of *TaClpB1* and *TaClpD2* was relatively higher and ubiquitously detected in all the tissues and developmental stages as compared to expression level of *TaClpB2, TaClpB3, TaClpB5, TaClpC1*, and *TaClpD1* (Figures 4A,B). *TaClpB4* showed highest expression in flag leaf while *TaClpD1* was highly expressed in leaf and flag leaf (Figure 4A).

**Table 3:** **Digital expression analysis**.

**Genes**	**NCBI UniGene Id**	**EST and cDNA source from various wheat tissues**					**No of ESTs available in NCBI**	**No of mRNA seq available in NCBI**
		**Leaf**	**Root**	**Stem**	**Inflorescence**	**Developing seed**		
*TaClpB1*	Ta.54573	✓		✓	✓	✓	400	41
*TaClpB2*	Ta.256	✓	✓		✓	✓	24	8
*TaClpB3*	Ta.261	✓		✓	✓	✓	15	14
*TaClpB4*	Ta.51477	✓					4	3
*TaClpB5*	Ta.48427	✓	✓		✓	✓	41	19
*TaClpC1*	Ta.49781	✓	✓	✓	✓	✓	293	3
*TaClpC2*	Ta.68721				✓			9
*TaClpD1*	Ta.54390	✓	✓	✓	✓	✓	220	28
*TaClpD2*	Ta.48556	✓	✓	✓	✓	✓	23	27

*NCBIUniGene ID was used to retrieve the respective Clp gene ESTs sequence information along with their respective cDNA library information from NCBI UniGene database (✓, Expressed; blank, not expressed)*.

**Figure 5:**
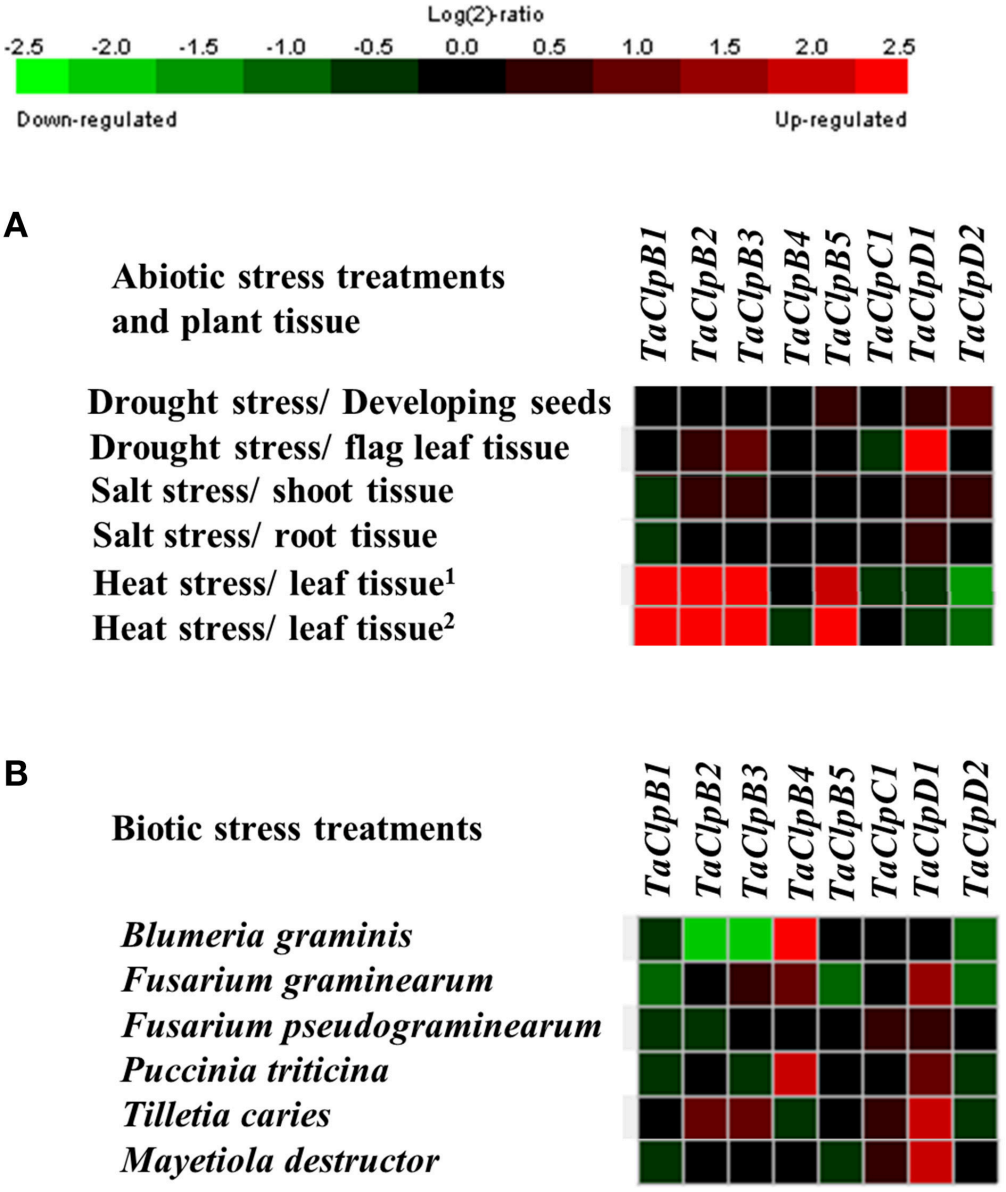
**Array based expression of ***TaClp*** genes under stress conditions. (A)** Expression of *Clp* genes under abiotic stress conditions. ^1^Expression profile of *TaClp* genes in heat sensitive genotype Chinese spring. ^2^Expression profile of *TaClp* genes in heat tolerant genotype TAM. **(B)** Expression of *Clp* genes under biotic stress conditions. The expression levels of *TaClp* genes are presented as heat maps generated using meta-analysis tool at Genevestigator (V3) http://www.genevestigator.ethz.ch. Details of wheat abiotic and biotic array experiments used in this study are given in Supplementary Table S2.

The array based expression analysis also revealed that *ClpB-cyt* genes (*TaClpB2* and *TaClpB3*) and *ClpB-m* (*TaClpB5*) transcripts were highly upregulated in leaf tissues under high temperature stress conditions in both heat-tolerant and heat-sensitive wheat genotypes (Figure 5A). *TaClpD1* expression was increased during drought stress in flag leaf tissues (Figure 5A). Two genes, *TaClpB4* and *TaClpD1* were found to be most responsive to biotic stresses analyzed in this study. More than two fold increase in expression of *TaClpB4* was observed in response to fungal pathogen *Blumeria graminis* and *Puccinia triticina* treatments (Figure 5B) while *TaClpD1* was highly upregulated by *Tilletia caries* and *Mayetiola destructor* treatments (Figure 5B). In contrast, *TaClpB2* and *TaClpB3* expression levels were decreased by more than 2.5 fold in response to fungal pathogen *B. graminis* treatment (Figure 5B).

### qRT-PCR expression analysis of wheat class I Clps

**Figure 6:**
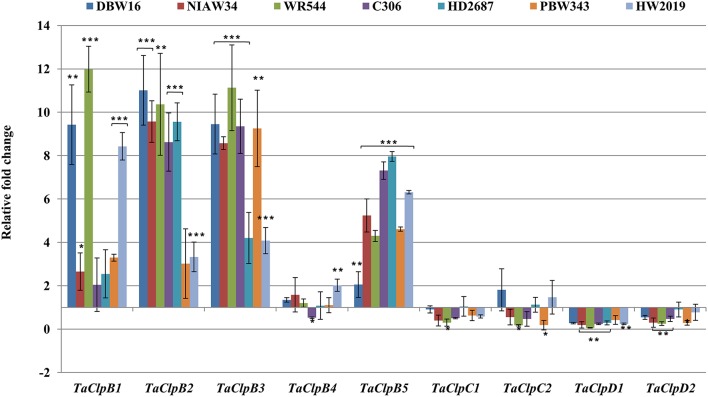
**Expression levels of ***TaClp***genes under high temperature stress**. High temperature stress was imposed to 15 days old seedlings at 42 ± 2°C for 3 h. Error bar represents standard deviation of three biological replicates. *p* value is calculated using two tailed unpaired *t*-test, ^*^p < 0.05, ^**^p < 0.01, ^***^p < 0.001.

**Figure 7:**
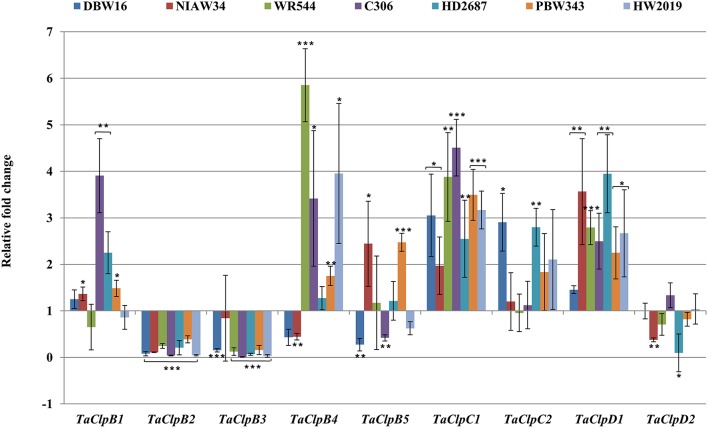
**Expression levels of ***TaClp*** genes under cold stress**. Cold stress was imposed to 15 days old seedlings at 2 ± 2°C for 3 h. Error bar represents standard deviation of three biological replicates. *p* value is calculated using two tailed unpaired *t*-test, ^*^p < 0.05, ^**^p < 0.01, ^***^p < 0.001.

**Figure 8:**
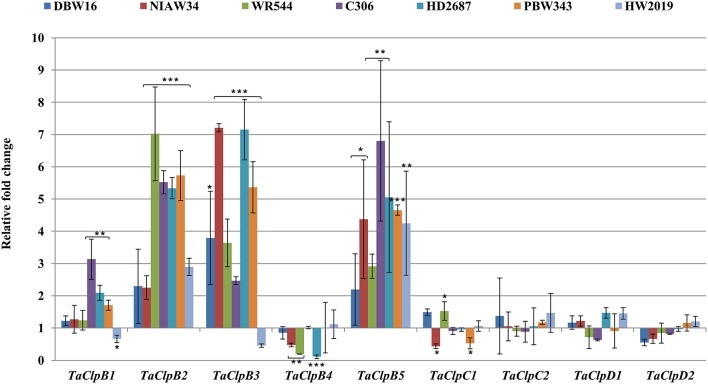
**Expression levels of ***TaClp*** genes under oxidative stress**. For oxidative stress, 15 days old seedlings were treated with 10 mM H_2_O_2_ and kept in dark conditions for 6 h. Error bar represents standard deviation of three biological replicates. *p* value is calculated using two tailed unpaired *t*-test, ^*^p < 0.05, ^**^p < 0.01, ^***^p < 0.001.

**Figure 9:**
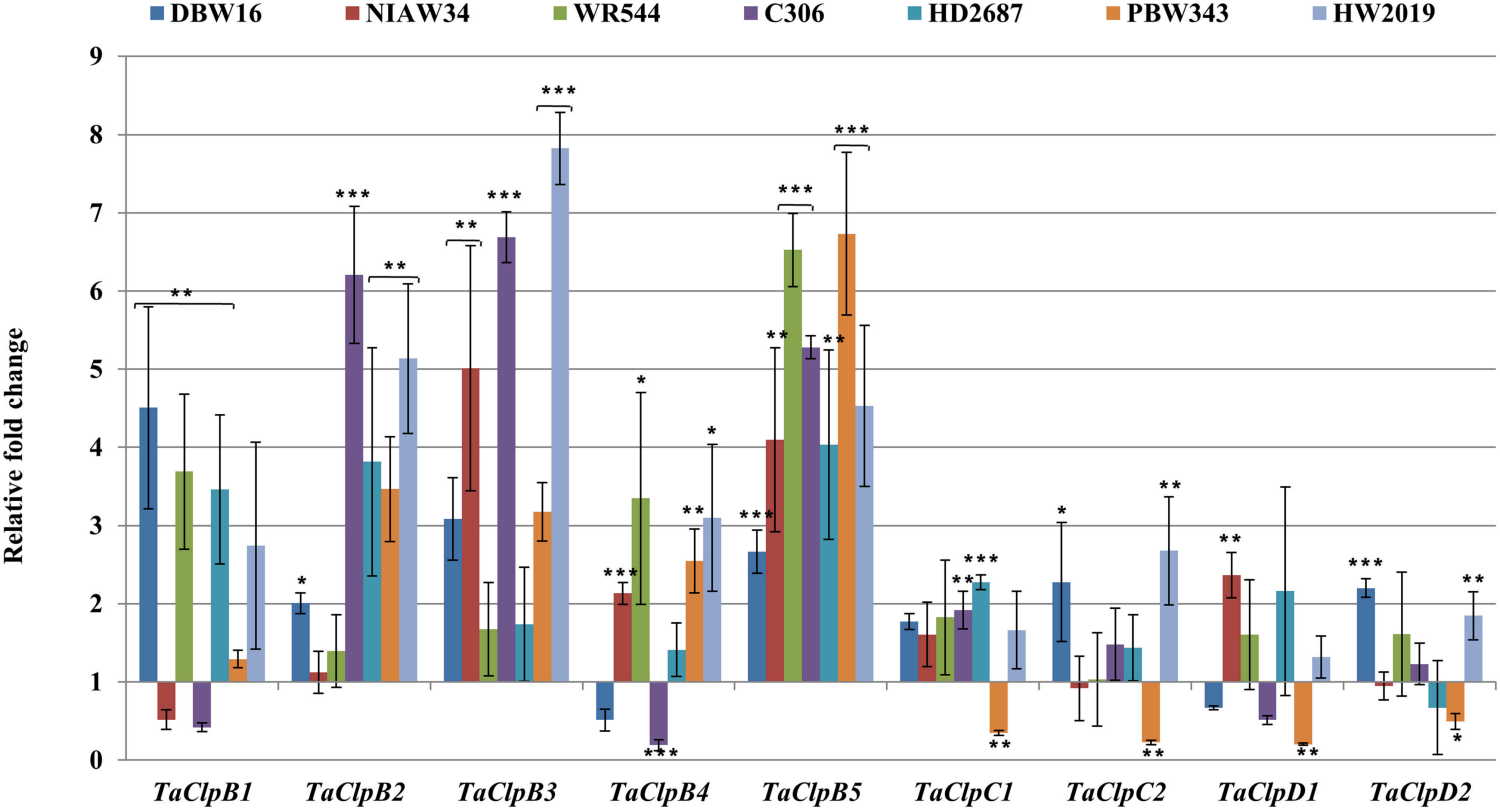
**Expression levels of ***TaClp*** genes under salt stress**. Salt stress was imposed to 15 days old seedlingsby saturating the cotton bed with 150 MmNaCl for 6 h. Error bar represents standard deviation of three biological replicates. *p* value is calculated using two tailed unpaired *t*-test, ^*^p < 0.05, ^**^p < 0.01, ^***^p < 0.001.

In crop plants, class I ClpATPases are expressed in most of the tissues, at different developmental stages and get induced under various abiotic stress conditions (Keeler et al., 2000; Singh and Grover, 2010; Singh et al., 2010). To investigate the response of *TaClp* genes to different abiotic stresses, a comprehensive analysis of their expression was performed in 15 days old seedlings of seven wheat genotypes (*T. aestivium* cvs DBW16, NIAW34, WR544, C306, HD2687, PBW343, and HW2019) differing in thermotolerance. These genotypes were subjected to various abiotic stress conditions such as, high temperature, cold, oxidative, and salt stress (Figures 6–**9**).

The expression levels of *TaClpB1, TaClpB2, TaClpB3*, and *TaClpB5* were either unchanged or up regulated under high temperature stress in all the wheat genotypes (Figure 6). However, the level of expression varied among the genotypes. There was 8–12 fold increase in expression of *TaClpB1* in wheat genotypes, DBW16, WR544, and HW2019 whereas the genotypes NIAW34, C306, HD2687 showed two fold or lesser increase in expression of *TaClpB1* (Figure 6). Similarly 8–11 fold increased in expression of *TaClpB2* and *TaClpB3* genes was recorded in five out of seven genotypes under high temperature stress condition (Figure 6). *TaClpB5* expression was enhanced by 4–8 folds in all the genotypes except DBW16 (Figure 6). High temperature stress did not change the expression pattern of *TaClpB4, TaClpC1, TaClpC2, TaClpD1*, and *TaClpD2* genes among wheat genotypes (Figure 6).

*TaClpC1* and *TaClpD1* were upregulated while TaClpB2 and TaClpB3 were significantly downregulated in all the genotypes under cold stress conditions (Figure 7). Expression of *TaClpB1, TaClpB4*, and *TaClpB5* were upregulated in some genotypes and downregulated in other genotypes (Figure 7). The expression profile of *TaClpB2, TaClpB3*, and *TaClpB5* under oxidative stress was similar to that recorded under high temperature stress. Expression of these three genes was significantly enhanced in all wheat genotypes except for down regulation of *TaClpB3* in HW2019 (Figure 8). *TaClpB1* expression was either unchanged as in DBW16, NIAW34, and WR544 or showed induction of 1.7–3 fold in C306, HD2687, and PBW343 (Figure 8). There was no significant difference in the expression of *TaClpC1, TaClpC2, TaClpD1*, and *TaClpD2* under oxidative stress in all the genotypes while *TaClpB4* was significantly down regulated in NIAW34, WR544, and HD2687 (Figure 8).

All class I *TaClp* genes showed upregulation at least in some of the genotypes under salt stress. The level of induction by salt stress was higher (up to eight fold) for the *TaClpB* genes as compared to that of *TaClpC* and *TaClpD* genes (up to 2.7 fold) (Figure 9). Interestingly *TaClpC* and *TaClpD* genes were consistently downregulated in stress susceptible wheat genotype PBW343.

## Discussion

Class I ClpATPases (ClpB/HSP100, ClpC, and ClpD) function as molecular chaperones and play vital role in thermotolerance of plants. Arabidopsis and rice genomes encode 6 and 9 Class I Clps, respectively (Zheng et al., 2002; Singh et al., 2010). As in case of rice, we identified 9 Class I *ClpATPases* genes in wheat which include five *ClpB* genes, two *ClpC* genes, and two *ClpD* genes (Table 1). Multiple sequence alignment and phylogenetic analyses of wheat, Arabidopsis, rice, maize, sorghum, brachypodium, and setaria Clp sequences revealed that the Class I ATPases are highly conserved across the grass genome (Figure 2). The phylogenetic tree branched into three major clades corresponding to ClpB, ClpC, and ClpD protein family, respectively (Figure 2). The ClpB proteins were categorized into cytoplasmic, mitochondrial and chloroplastic isoforms based on their sub-cellular localization predicted by TargetP and WoLF PSORT tool (Supplementary Table S1). The Class I ATPase protein family is highly conserved molecular chaperones in most organisms from bacteria to plants (Trösch et al., 2015). It was evident from the similar exon-intron structures of *TaClpB5, ClpC1, ClpD1*, and *ClpD2* family genes of Arabidopsis and grass genomes (Figure 3). *TaClpB5* family genes contain 10 exons, and *ClpD1* and *ClpD2* family genes contain 12 exons each (Figure 3).

The array based expression analysis revealed significant spatio-temporal transcriptional regulation of *TaClps* by developmental and stress cues (Figures 4A,B; 5A,B). In addition to chaperone activity, ClpATPase proteins are also involved in various cellular process related to plant growth and development (Trösch et al., 2015). The ubiquitous presence of *TaClpB1* and *TaClpD2* and their abundance in most of the tissues and developmental stages indicate the significance of these genes in growth and development of wheat. The higher expression of *TaClpB2* and *TaClpB3* in embryo, endosperm, caryopsis and root reflect their specific role during seed development and in roots (Figure 4A). These observations are further corroborated in array analysis which exhibited higher expression levels of *TaClpB2* during milk, dough, and ripening stages of wheat grain development (Figure 4B). In Arabidopsis, loss of function of *AtClpC1* resulted in reduced plant growth, lowered protein translocation and chloroplast development (Constan et al., 2004; Olinares et al., 2011). In this study, the expression of *TaClpC1* was not only found in green tissues but also in non-green tissues such as root and dough stage of grain development (Figures 4A,B) suggesting its potential role beyond chloroplast development and function in wheat.

Cytoplasm localized ClpBs (ClpB-cyt) act as molecular chaperones, and mediate disaggregation of denatured proteins during high temperature stress (Singh and Grover, 2010). In Arabidopsis (Lee et al., 2007), rice (Singh et al., 2010), maize (Nieto-Sotelo et al., 2002), and Lima bean (Keeler et al., 2000), *ClpB-cyt* are involved in both basal and acquired thermotolerance. Arabidopsis *ClpB-cyt, ClpB-p*, and *ClpB-m* transcripts accumulate dramatically at high temperature stress (Lee et al., 2007). Transgenic tomato lines with antisense suppression of chloroplast localized *ClpB-P* (*LeHSP100/ClpB*) failed to recover from heat shock treatment (Yang et al., 2006). Overexpression of Arabidopsis *ClpB-cyt* (*AtHSP101*) in an *indica* rice variety Pusa basmati 1 resulted in increased basal thermotolerance in transgenic rice (Katiyar-Agarwal et al., 2003). Wheat *ClpB-cyt* genes, *TaHSP101B* and *TaHSP101C*, were found to be induced by high temperature stress, dehydration and ABA, but not affected by chilling or wounding (Campbell et al., 2001). Expression of *TdHSP101B* and *TdHSP101C* were increased under high temperature stress in *Triticum durum* (Gullì et al., 2007; Rampino et al., 2009, 2012). In maize, *ClpB-cyt* null mutants were defective in both induced and basal thermotolerance (Nieto-Sotelo et al., 2002). Our array based analysis also showed that wheat *ClpB-p* (*TaClpB1*), and *ClpB-cyt* (*TaClpB2* and *TaClpB3*), and *ClpB-m* (*TaClpB5*) genes were upregulated under high temperature stress conditions in both heat-tolerant and heat-sensitive genotypes (Figure 5A). We further confirmed this by real-time RT-PCR expression analysis wherein high temperature stress was found to upregulate the expression of *ClpB-p* (*TaClpB1*), *ClpB-cyt* (*TaClpB2* and *TaClpB3*), and *ClpB-m* (*TaClpB5*) in all wheat genotypes (Figure 6). This suggests their potential role in imparting basal thermotolerance to wheat genotypes. In contrast, *TaClpD2* showed downregulation in both array based analysis and qRT-PCR analysis (Figures 5A, 6). Sumesh et al. (2008) observed higher amount of TaClpB/HSP100 content at elevated temperature in a relatively tolerant wheat variety. In our study, high temperature stress enhanced the expression of *TaClpB2* and *TaClpB3* genes in all the genotypes. However, *TaClpB2* and *TaClpB3* expression levels were higher in the all four thermotolerant genotypes (DBW16, NIAW34, WR544, and C306) and lower in at least two out of three sensitive genotypes (Figure 6). Among thermotolerant genotypes, DBW16 and WR544 displayed higher expression of *ClpB-p* (*TaClpB1*), and *ClpB-cyt* (*TaClpB2* and *TaClpB3*) genes than C306 and NIAW34 (Figure 6). All the genotypes showed 2–8 fold increase in *ClpB-m* (*TaClpB5*) expression under heat stress (Figure 6). In Arabidopsis, all the *ClpB* transcripts were upregulated by heat while *ClpC and ClpD* were not regulated by heat. Moreover, the T-DNA mutants of *ClpB-p* and *ClpB-m* were not defective in acquired thermotolerance as compared to heat sensitive null mutant of cytosolic/nuclear *AtHsp101* (*ClpB-cyt*) (Lee et al., 2007). In our study also transcript levels of *ClpB-cyt* (*TaClpB2* and *TaClpB3*) were up regulated in four of the thermotolerant genotypes which emphasizes the critical role of *ClpB-cyt* in thermotolerance (Figure 6). The relatively heat sensitive genotype HD2687 and PBW343 demonstrated higher *TaClpB2* and *TaClpB3* expression levels, respectively (Figure 6). This suggests that simultaneous expression of both the *ClpB-cyt* genes (*TaClpB2* and *TaClpB3*) might be required for better thermotolerance. Thus, TaClpB/HSP100 might be playing important role than *ClpB-p* (*TaClpB1*) and *ClpB-m* (*TaClpB5*) genes in imparting thermotolerance in wheat.

Cold stress is known to upregulate rice *ClpC* transcripts (Singh et al., 2010) and protein (Cui et al., 2005). ClpC1 is associated with the chloroplast protein translocation machinery and might play important role in importing stress responsive protein under stress conditions (Adam et al., 2006; Kovacheva et al., 2007). *AtClpD* expression was increased during cold stress (Zheng et al., 2002). *ZmHsp101* (ortholog of *TaClpB2* and *TaClpB3*) expression was increased only during high temperature stress not under cold, osmotic and ABA induced stress conditions (Nieto-Sotelo et al., 1999). In our study also cold stress induced the expression of *TaClpC1* and *TaClpD1* (Figure 7), while the expression of *TaClpB2* and *TaClpB3* was enhanced by heat stress but downregulated by cold stress in most of the wheat genotypes (Figure 7). However, we also observed the induced expression of *TaClpB1* in C306 and HD2687, *TaClpB4* expression in WR544, HW2019 and C306, *TaClpB5* expression in NIAW34 and PBW343, and *TaClpC2* expression in DBW16, HD2687, PBW343, and HW2019 under cold stress (Figure 7). These results suggest that *TaClpC1* and *TaClpD1* may play specific protective role under low temperature stress, while *TaClpB2* and *TaClpB3* may be important specifically for high temperature stress response of wheat. This also highlights the highly conserved role of these proteins across plant species. *OsClpB-cyt* and *OsClpB-m* expression was increased under oxidative stress treatment (Singh et al., 2010). In our study also oxidative stress upregulated the expression of *Clp-cyt* (*TaClpB2* and *TaClpB3)* and *Clp-m* (*TaClpB5)* in all the genotypes (except for *TaClpB3* in HW2019) (Figure 8). *TaClpB1* expression was induced in C306 and HD2687 under oxidative stress (Figure 8). In rice, oxidative stress induced the expression of *OsClpC2, OsClpD1*, and *OsClpD2* (Singh et al., 2010). *Arabidopsis ClpD* also showed upregulation under short-term severe oxidative stress (Zheng et al., 2002). However, we did not find significant difference in the expression levels of *TaClpC1, TaClpC2, TaClpD1*, and *TaClpD2* under oxidative stress in the wheat genotypes (Figure 8).

Drought and salt stress inducible *AtClpD* was shown to impart drought tolerance to Arabidopsis (Nakashima et al., 1997; Tran et al., 2004). We also found that drought stress upregulates the expression of *TaClpD1* (Figure 5A). In rice, ClpB proteins were enhanced during salt stress (Pareek et al., 1995). *AtClpC* expression was increased under short-term severe salt stress (Zheng et al., 2002). In this study, *TaClpB* genes were upregulated in most of the wheat genotypes under salt stress (Figure 9). However, there was no distinct pattern of expression difference between thermotolerant and susceptible genotypes. All the genotypes showed induction of *TaClpB5* expression in the range of ~2.75–6.75 fold under salt stress (Figure 9). *TaClpB1* expression was induced in DBW16, WR544, HD2687, and HW2019 whereas, *TaClpB2* expression was induced in all the genotypes (except in NIAW34 andWR544). *TaClpB4* expression was induced in NIAW34, WR544, PBW343, and HW2019 (Figure 9). *TaClpC1* was induced in all the genotypes (except in PBW343), whereas *TaClpC1* and *TaClpD2* expression was induced in DBW 16 and HW2019 (Figure 9). *TaClpD1* was induced in NIAW34 and HD2687 (Figure 9). One of the gene encoding plastid Clp, *TaClpD2* was upregulated only under salt stress (Figure 9). In our study, the *TaClps* were differentially expressed among the genotypes under abiotic stress conditions (Figures 6–9). Further studies on mining the regulatory sequences of *TaClps* in contrasting genotypes and their regulatory mechanisms will enhance the understanding of *TaClps* in wheat.

In tomato, prolonged infection of tomato yellow leaf curl virus resulted in decline in the amounts of HSPs, which was more pronounced in susceptible than in resistant plants (Gorovits and Czosnek, 2007). In our study, Clps were found to be regulated by various biotic stresses (Figure 5B). *TaClpD1* was upregulated by both fungal pathogens and insect pests, while *TaClpB4* was highly upregulated under bacterial and fungal pathogen treatments (Figure 5B). These results suggest potential biotic-stress specific roles of *Clp* genes in wheat.

## Conclusion

This study elucidated the genomic organization of *TaClps*, evolutionary relationship with the grass orthologous and developmental and stress regulated expression of *TaClp* gene family in wheat. We identified nine class I Clp proteins in wheat and showed high conservation of ClpB/Hsp100, ClpC and ClpD proteins across grass genomes. We noted that several *TaClps* genes are expressed constitutively in vegetative tissues and various developmental stages. Through wheat array transcript profiling and qRT-PCR analysis, we have shown that the class I *Clp* genes are differentially regulated by abiotic stresses. High temperature and oxidative stresses upregulated *TaClpB1* (*ClpB-p*), *TaClpB2* and *TaClpB3* (*ClpB-cyt*), and *TaClpB5* (*ClpB-m*) expression in most of the genotypes except that of *TaClpB3* in HW2019 under oxidative stress. Cold stress specifically upregulated *TaClpC* and *TaClpD1* genes. The *TaClpB5* gene for mitochondrial Clp showed upregulation under heat, salt and oxidative stresses. The differential expression regulation suggests a distinct role of *TaClpB2, TaClpB3*, and *TaClpB5* in high temperature stress response, and *TaClpC1, TaClpC2*, and *TaClpD1* in low temperature stress response of wheat. Some of the *TaClps* showed considerable upregulation under fungal pathogen treatment and insect pest attack. Our results showed wheat *ClpB/Hsp100, ClpC*, and *ClpD* genes play distinct roles in plant growth, development and response to abiotic and biotic stress conditions.

## Author contributions

SM, VC, and KB conceived the idea and designed the experiments. SM performed all the experiments, analyse the data, and drafted the manuscript. MD contributed to data analysis. MD, VC, and KB edited the manuscript. All authors have read and approved the final manuscript.

### Conflict of interest statement

The authors declare that the research was conducted in the absence of any commercial or financial relationships that could be construed as a potential conflict of interest.

## Abbreviations

Clp: Caseinolytic protease
HSP: Heat shock proteins
ClpB-cyt: Cytoplasmic ClpB
ClpB-p: Chloroplastic ClpB
ClpB-m: Mitochondrial ClpB.
