# Supplementary material for: Differential Regulation of Genes Coding for Organelle and Cytosolic ClpATPases under Biotic and Abiotic Stresses in Wheat
Source: Front Plant Sci. 2016 Jun 28;7:929. doi: 10.3389/fpls.2016.00929 (PMC4923199; doi:10.3389/fpls.2016.00929)
Supplement: Supplementary file 1 [file Table1.DOCX]

**Supplementary Table S1. List of class I Clp ATPase proteins from Arabidopsis, Brachypodium, Maize, Rice, Sorghum and Setaria.** In the enzyme IDs, At indicates *Arabidopsis thaliana* LOC_Os indicates *Oryza sativa*, Sb indicates *Sorghum bicolor*, GRMZM indicates *Zea mays*, Bradi indicates *Brachypodiumdistachyon*and Si indicates *Setariaitalica*. aa- amino acids; pI- isoelectric point. Mw- Molecular weight; The subcellular localization of Clps were predicted through WoLF PSORT tool and TargetP 1.1 server.

| **Gene** | **Protein ID** | **Size (aa)** | **Mw**  **(K Da)** | **pI** | **Sub cellular localization** |
| --- | --- | --- | --- | --- | --- |
| *ClpB* | At1g74310 | 911 | 101.3 | 5.81 | Cytosol |
|  | At2g25140 | 964 | 108.66 | 6.54 | Mitochondria |
|  | At5g15450 | 968 | 108.94 | 5.93 | Chloroplast |
|  | Bradi2g19540.1 | 912 | 100.77 | 5.89 | Cytosol |
|  | Bradi2g49660.1 | 913 | 101.22 | 5.86 | Cytosol |
|  | Bradi3g06107.1 | 987 | 109.66 | 6.59 | Mitochondria |
|  | Bradi1g16190.1 | 970 | 108.51 | 6.50 | Chloroplast |
|  | GRMZM2G360681_T01 | 912 | 101.12 | 5.84 | Cytosol |
|  | GRMZM2G060561_T02 | 988 | 109.55 | 6.12 | Mitochondria |
|  | GRMZM2G162968_T01 | 974 | 108.77 | 7.62 | Chloroplast |
|  | LOC_Os05g44340.1 | 912 | 100.9 | 5.90 | Cytosol |
|  | LOC_Os02g08490.1 | 983 | 109.25 | 6.80 | Mitochondria |
|  | LOC_Os03g31300.1 | 978 | 108.99 | 6.25 | Chloroplast |
|  | Sb09g025900.1 | 913 | 101.19 | 5.75 | Cytosol |
|  | Sb03g034390.1 | 915 | 101.53 | 6.20 | Cytosol |
|  | Sb04g005570.1 | 990 | 109.77 | 6.38 | Mitochondria |
|  | Sb01g032210.1 | 983 | 109.41 | 6.88 | Chloroplast |
|  | Si021144m | 913 | 101.02 | 5.80 | Cytosol |
|  | Si000232m | 914 | 101.03 | 5.85 | Cytosol |
|  | Si016214m | 989 | 109.42 | 6.26 | Mitochondria |
|  | Si034086m | 977 | 108.84 | 6.67 | Chloroplast |
| *ClpC* | At3g48870 | 952 | 105.74 | 6.06 | Chloroplast |
|  | At5g50920 | 929 | 103.45 | 6.36 | Chloroplast |
|  | LOC_Os12g12850.1 | 919 | 102.02 | 6.62 | Chloroplast |
|  | LOC_Os04g32560.1 | 918 | 101.80 | 6.14 | Chloroplast |
|  | LOC_Os11g16590.1 | 932 | 100.88 | 8.31 | Chloroplast / Mitochondria |
|  | LOC_Os11g16770.1 | 918 | 100.99 | 9.47 | Plasma membrane |
|  | Bradi3g44340.3 | 921 | 102.02 | 6.14 | Chloroplast |
|  | Bradi4g20470.1 | 943 | 103.21 | 6.10 | Chloroplast |
|  | Bradi4g39880.1 | 920 | 101.74 | 6.62 | Chloroplast |
|  | GRMZM2G009443_T01 | 921 | 102.04 | 6.24 | Chloroplast |
|  | GRMZM2G123922_T01 | 921 | 102.07 | 6.23 | Chloroplast |
|  | Sb08g007750.1 | 921 | 102.07 | 6.50 | Chloroplast |
|  | Sb06g014590.1 | 921 | 102.14 | 6.32 | Chloroplast |
|  | Si021139m | 921 | 102.09 | 6.62 | Chloroplast |
|  | Si009304m | 921 | 102.20 | 6.23 | Chloroplast |
|  | Si009306m | 918 | 101.76 | 6.26 | Chloroplast |
| *ClpD* | At5g51070 | 945 | 103.23 | 5.89 | Chloroplast |
|  | Bradi3g44640.1 | 944 | 102.27 | 6.60 | Chloroplast |
|  | Bradi5g08920.1 | 942 | 102.24 | 9.00 | Chloroplast |
|  | LOC_Os02g32520.1 | 938 | 101.88 | 6.78 | Chloroplast |
|  | LOC_Os04g33210.1 | 945 | 102.53 | 8.47 | Chloroplast |
|  | GRMZM2G172230_T01 | 947 | 102.37 | 6.21 | Chloroplast |
|  | GRMZM2G149567_T01 | 932 | 101.31 | 8.60 | Chloroplast |
|  | Sb04g021410.1 | 957 | 103.67 | 6.67 | Chloroplast |
|  | Sb06g015220.1 | 939 | 102.10 | 8.68 | Chloroplast |
|  | Si016236m | 942 | 102.32 | 6.57 | Chloroplast |
|  | Si009283m | 942 | 102.49 | 8.50 | Chloroplast |

**Supplementary Table S2.List of Wheat array experiments used in this study**

| **GenevestigatorExperiment ID** | **NCBI GEO Accession/ EBI ArrayExpress ID** | **Title** | **Reference** |
| --- | --- | --- | --- |
| TA-00009 | [GSE12936](http://www.ncbi.nlm.nih.gov/geo/query/acc.cgi?acc=GSE12936) | Transcriptomic analysis of the effect of silicon on wheat plants infected or uninfected with powdery mildew | (Chain et al., 2009) |
| TA-00013 | [GSE21386](http://www.ncbi.nlm.nih.gov/geo/query/acc.cgi?acc=GSE21386) | RNA profiling of Fusarium head blight-resistant wheat addition lines containing the *Thinopyrum elongatum* chromosome 7E | (Wang et al., 2010) |
| TA-00014 | [GSE22080](http://www.ncbi.nlm.nih.gov/geo/query/acc.cgi?acc=GSE22080+) | Pleiotropic expression of endogenous genes upon fungus infection of wheat plants containing anti-fungal transgenes | unpublished |
| TA-00039 | [GSE34445](http://www.ncbi.nlm.nih.gov/geo/query/acc.cgi?acc=GSE34445) | Expression data from wheat following Hessian fly larval attack | (Zhu et al., 2012) |
| TA-00005 | [GSE9915](http://www.ncbi.nlm.nih.gov/geo/query/acc.cgi?acc=GSE9915) | Transcript profiling of Lr1- and Lr34-mediated leaf rust resistance in wheat | (Bolton et al., 2008) |
| TA-00010 | [GSE13660](http://www.ncbi.nlm.nih.gov/geo/query/acc.cgi?acc=GSE13660) | Gene expression analysis of the wheat response to infection by *Fusarium pseudograminearum* | (Desmond et al., 2008) |
| TA-00028 | [GSE31762](http://www.ncbi.nlm.nih.gov/geo/query/acc.cgi?acc=GSE31762) | Wild emmer wheat comparison of drought resistant vs. susceptible genotypes under terminal drought | (Krugman et al., 2010) |
| TA-00015 | [E-MEXP-1193](http://www.ebi.ac.uk/arrayexpress/experiments/E-MEXP-1193/) | Transcription profiling time series of wheat cv. Hereward grown under control, hot, dry and hot and dry conditions to illustrate the importance of developmental context in interpretation | (Wan et al., 2008) |
| TA-00021 | [E-MEXP-971](http://www.ebi.ac.uk/arrayexpress/experiments/E-MEXP-971/) | Transcription profiling of two highly salt-tolerant wheat lines, their parental lines, and a salt-sensitive line in salt stress and control growth conditions | (Mott and Wang, 2007) |
| TA-00030 | [E-MEXP-1488](http://www.ebi.ac.uk/arrayexpress/experiments/E-MEXP-1488/) | Transcription profiling of wild emmer wheat plants with different drought stress responses grown in hydroponics and shock-drought stressed for 4 and 8 hours | (Ergen et al., 2009) |
| TA-00019 | [E-MEXP-1523](http://www.ebi.ac.uk/arrayexpress/experiments/E-MEXP-1523/) | Transcription profiling of heat tolerant and susceptible strains of wheat after exposure to heat stress | (Qin et al., 2008) |

**Supplementary Table S3. List of primer sequence used in this study**

| **GENE** | **Forward primer sequence** | **Reverse primer sequence** |
| --- | --- | --- |
| *TaClpB1* | GTGCAATGGATGCTGGCAATCT | ATACACTTGTTGGAAGCGGCGT |
| *TaClpB2* | ATCCTGTTCGACGAGGTGGAGAA | TGGAGGTCATGATGATCACGGTGT |
| *TaClpB3* | CAGATGAAAGATGTGGCAGTCCGT | AGACCGGATCGTAAGACAGTGACA |
| *TaClpB4* | TAAGCATACAGGACCGTGCATTGG | CTTCCTCTCGAGGCTGTCAATCTC |
| *TaClpB5* | CAAACAGAACGACAGAGTAAG | CCTTAATACGGATTGACATGAG |
| *TaClpC1* | ATTAGCCGAGGAGGGAAAGCTAGA | TCAGGCACATCGCCACTAGAAATG |
| *TaClpC2* | CGGCAGCCATTGTACTCAAGAACT | CTGGCACCGACAGTTTGATCTTCA |
| *TaClpD1* | GGCAAAGCGGATAATGTCGCTAGA | GCTAATATCAAGGCCAGCACCCTTAC |
| *TaClpD2* | TCTGGATGAGCACCGTTTGCAT | ACTGCCGCATTGATGCCTTCTA |
| *Ubiquitin* | AGCGCAAGAAGAAGACGTACACCA | TAAGCCTGCTGGTTGTAGACGTAG |

**References**

Bolton, M. D., Kolmer, J. A., Xu, W. W., and Garvin, D. F. (2008). Lr34-Mediated Leaf Rust Resistance in Wheat: Transcript Profiling Reveals a High Energetic Demand Supported by Transient Recruitment of Multiple Metabolic Pathways. *Mol. Plant-Microbe Interact.* 21, 1515–1527. doi:10.1094/MPMI-21-12-1515.

Chain, F., Côté-Beaulieu, C., Belzile, F., Menzies, J. G., and Bélanger, R. R. (2009). A Comprehensive Transcriptomic Analysis of the Effect of Silicon on Wheat Plants Under Control and Pathogen Stress Conditions. *Mol. Plant-Microbe Interact.* 22, 1323–1330. doi:10.1094/MPMI-22-11-1323.

Desmond, O. J., Manners, J. M., Schenk, P. M., Maclean, D. J., and Kazan, K. (2008). Gene expression analysis of the wheat response to infection by Fusarium pseudograminearum. *Physiol. Mol. Plant Pathol.* 73, 40–47. doi:10.1016/j.pmpp.2008.12.001.

Ergen, N. Z., Thimmapuram, J., Bohnert, H. J., and Budak, H. (2009). Transcriptome pathways unique to dehydration tolerant relatives of modern wheat. *Funct. Integr. Genomics* 9, 377–96. doi:10.1007/s10142-009-0123-1.

Krugman, T., Chagué, V., Peleg, Z., Balzergue, S., Just, J., Korol, A. B., Nevo, E., Saranga, Y., Chalhoub, B., and Fahima, T. (2010). Multilevel regulation and signalling processes associated with adaptation to terminal drought in wild emmer wheat. *Funct. Integr. Genomics* 10, 167–86. doi:10.1007/s10142-010-0166-3.

Mott, I. W., and Wang, R. R.-C. (2007). Comparative transcriptome analysis of salt-tolerant wheat germplasm lines using wheat genome arrays. *Plant Sci.* 173, 327–339. doi:10.1016/j.plantsci.2007.06.005.

Qin, D., Wu, H., Peng, H., Yao, Y., Ni, Z., Li, Z., Zhou, C., and Sun, Q. (2008). Heat stress-responsive transcriptome analysis in heat susceptible and tolerant wheat (Triticum aestivum L.) by using Wheat Genome Array. *BMC Genomics* 9, 432. doi:10.1186/1471-2164-9-432.

Wan, Y., Poole, R. L., Huttly, A. K., Toscano-Underwood, C., Feeney, K., Welham, S., Gooding, M. J., Mills, C., Edwards, K. J., Shewry, P. R., et al. (2008). Transcriptome analysis of grain development in hexaploid wheat. *BMC Genomics* 9, 121. doi:10.1186/1471-2164-9-121.

Wang, J.-R., Wang, L., Gulden, S., Rocheleau, H., Balcerzak, M., Hattori, J., Cao, W., Han, F., Zheng, Y.-L., Fedak, G., et al. (2010). RNA profiling of fusarium head blight-resistant wheat addition lines containing the Thinopyrum elongatum chromosome 7E. *Can. J. Plant Pathol.* 32, 188–214. doi:10.1080/07060661003740512.

Zhu, L., Liu, X., Wang, H., Khajuria, C., Reese, J. C., Whitworth, R. J., Welti, R., and Chen, M.-S. (2012). Rapid Mobilization of Membrane Lipids in Wheat Leaf Sheaths During Incompatible Interactions with Hessian Fly. *Mol. Plant-Microbe Interact.* 25, 920–930. doi:10.1094/MPMI-01-12-0022-R.
