# Supplementary material for: Differential Regulation of Genes Coding for Organelle and Cytosolic ClpATPases under Biotic and Abiotic Stresses in Wheat
Source: Front Plant Sci. 2016 Jun 28;7:929. doi: 10.3389/fpls.2016.00929 (PMC4923199; doi:10.3389/fpls.2016.00929)
Supplement: Supplementary file 2 [file Image1.PDF]

```
TaClpB1                 MATAPPPALAADLHIS------SYPAAAPAPAVAAAAWGSSRRAAGPSST-------RVA  47
Bradi1g16190.1          --MAAAPPLTADFLVS------SSPAAAPAPVVA-AAWANRRGA-G-SSR-------CRA  42
LOC_Os03g31300.1        MAAAPPLAAGL-----------RPAMAAAQAPVVAAAWGVGARRGAALSSSARCRALRLS  49
Si034086m               MAAAPPL--SADALSFLPSA--AAPAAAAPTPVVAAAWGAARAAGSVRGKA----ALRMA  52
Sb01g032210.1           MAAAPPLSLSADVLSFLPSASASAPAAAAPTPVVAAAWGAARA-GAVRGKA----ALRTT  55
GRMZM2G162968_T01       MAAAPPL--SADVLSFLTSA------PAAPTPVVAAAWGAARA-GPVRGKA----ALRT-  46
                           *                       *    *. ***.         .

TaClpB1                 LSAARGRGR-LSPVVGT--GRPALSVRCNASSRDGRITQQEFTEMAWQSIVLAPEVAKES  104
Bradi1g16190.1          LRATRGRGW-LAPVVGR--TPRTLSVRCNASSRDGRITQQEFTEMAWQSIVLAPEVAKES  99
LOC_Os03g31300.1        RGGGGGRDGWVPPPVVGRMPPRTLSVRCAA--SNGRITQQEFTEMAWQSIVSSPEVAKES  107
Si034086m               R--R---GSGLAPVVGRRPRRPPLSVRCDATSRDGRITQQEFTEMAWQSIVSSPEVAKES  107
Sb01g032210.1           T--RGGRGGLLAPVVGGKPRRTPLSVRCNATSRDGRITQQEFTDMAWQAIVSSPEVAKES  113
GRMZM2G162968_T01       T--RGGRGGVLAPLVGRRPRRTPLSVRCNATSRDGRITQQEFTEMAWQAIVSSPEVAKES  104
                                  : * *        ***** *   :*********:****:** :*******

TaClpB1                 KHQIVETEHLMKSLLEQRNGLARRIFSKAGVDNTRLLDATEKFIQRQPKVLGEDPGSMLG  164
Bradi1g16190.1          KHQIVETEHLMKSLLEQRNGLARRIFLKAGVDNTKLLDATEKYIQRQPKVLGEDPGSMLG  159
LOC_Os03g31300.1        KHQIVETEHLMKSLLEQRNGLARRIFSKAGVDNTRLLDATEKFIQRQPKVLGEDPGSMLG  167
Si034086m               KHQIVETEHLMKSLLEQRNGLARRIFSKAGVDNTKLLDATEKFIQRQPKVLGEDPGSMLG  167
Sb01g032210.1           KHQIVETEHLMKSLLEQRNGLARRIFSKAGVDNTRLLDAAEKFIQRQPKVLGEEPGSMLG  173
GRMZM2G162968_T01       KHQIVETEHLMKSLLEQRNGLARRIFSKAGVDNTRLLDATKKFIQRQPKVLGEEPGSMLG  164
                        ************************** *******:****::*:**********:******

TaClpB1                 RDLEALIQRARNFKKEYGDSFVSVEHIVLGFADDKRFGRQLFKDFQITVESLKTAIESIR  224
Bradi1g16190.1          RDLEALIQRARDFKKEYGDSFVSVEHLVLGFADDKRFGRQLFKDFLITVKTLKSAIESIR  219
LOC_Os03g31300.1        RDLEALIQRARDFKKEYGDSFVSVEHLVLGFAEDKRFGRQLFKDFQITVQSLKTAIESIR  227
Si034086m               RDLEALIQRARDFKKEYGDSYVSVEHLVLGFAEDKRFGKQLFKDFQITVKTLKLAIESIR  227
Sb01g032210.1           RDLEALIQRARDFKKEYGDSYVSVEHLVLGFAEDKRFGKQLFKDFQVTVKTLKSAIESIR  233
GRMZM2G162968_T01       RDLEALIQRARDFKKEYGDSYVSVEHLVLGFAEDKRFGTQLFKDFQITVKTLKSAIESIR  224
                        ***********:********:*****:*****:***** ****** :**::** ******

TaClpB1                 GKQNVIDQDPEGKYEALDKYGKDLTAMARQGKLDPVIGRDDEIRRCIQILSRRTKNNPVL  284
Bradi1g16190.1          GKQNVIDQDPEGKYEALDKYGKDLTAMARQGKLDPVIGRDDEIRRCIQILSRRTKNNPVL  279
LOC_Os03g31300.1        GKQNVIDQDPEGKYEALDKYGKDLTAMARQGKLDPVIGRDDEIRRCIQILSRRTKNNPVL  287
Si034086m               GKQNVIDQDPEGKYEALEKYGKDLTAMARQGKLDPVIGRDDEIRRCIQILSRRTKNNPVL  287
Sb01g032210.1           GKQNVMDQDPEGKYEALEKYGKDLTAMARQGKLDPVIGRDDEIRRCIQILSRRTKNNPVL  293
GRMZM2G162968_T01       GKQNVIDQDPEGKYEALDKYGKDLTAMARQGKLDPVIGRDDEIRRCIQILSRRTKNNPVL  284
                        *****:***********:******************************************

TaClpB1                 IGEPGVGKTAIAEGLAQRIVQGDVPQALTNRRLITLDMGALIAGAKYRGEFEDRLKAVLK  344
Bradi1g16190.1          IGEPGVGKTAIAEGLAQRIVQGDVPQALTNRRLITLDMGALIAGAKYRGEFEDRLKAVLK  339
LOC_Os03g31300.1        IGEPGVGKTAIAEGLAQRIVQGDVPQALTNRRLIALDMGALIAGAKYRGEFEDRLKAVLK  347
Si034086m               IGEPGVGKTAIAEGLAQRIVQGDVPQALTNRRLIALDMGALIAGAKYRGEFEDRLKAVLK  347
Sb01g032210.1           IGEPGVGKTAIAEGLAQRIVQGDVPQALTNRRLIALDMGALIAGAKYRGEFEDRLKAVLK  353
GRMZM2G162968_T01       IGEPGVGKTAIAEGLAQRIVQGDVPQALTNRRLIALDMGALIAGAKYRGEFEDRLKAVLK  344
                        **********************************:*************************

TaClpB1                 EVTDSDGQVVLFIDEIHTVVGAGATSGAMDAGNLLKPMLGRGELRYIGATTLDEYRKYIE  404
Bradi1g16190.1          EVIDSDGQVILFIDEIHTVVGAGATSGAMDAGNLLKPMLGRGELRCIGATTLDEYRKYIE  399
LOC_Os03g31300.1        EVTDSDGQTILFIDEIHTVVGAGATNGAMDAGNLLKPMLGRGELRCIGATTLDEYRKYIE  407
Si034086m               EVTDSDGQTILFIDEIHTVVGAGATNGAMDAGNLLKPMLGRGELRCIGATTLDEYRKYIE  407
Sb01g032210.1           EVTDSDGQTILFIDEIHTVVGAGATNGAMDAGNLLKPMLGRGELRCIGATTLDEYRKYIE  413
GRMZM2G162968_T01       EVTDSDGQTILFIDEIHTVVGAGATNGAMDAGNLLKPMLGRGELRCIGATTLDEYRKYIE  404
                        ** *****.:***************.******************* **************

TaClpB1                 KDPALERRFQQVYVDQPTVEDTVSILRGLRERYELHHGVRISDSALVAAALLSDRYISGR  464
Bradi1g16190.1          KDPALERRFQQVYVDQPSVEDTVSILRGLRERYELHHGVRISDSALVAAAVLSDRYISGR  459
LOC_Os03g31300.1        KDPALERRFQQVYVDQPSVEDTISILRGLRERYELHHGVRISDSALVAAALLSDRYISGR  467
Si034086m               KDPALERRFQQVYVDQPSVEDTISILRGLRERYELHHGVRISDSALVAAAVLSDRYISGR  467
Sb01g032210.1           KDPALERRFQQVYVDQPSVEDTISILRGLRERYELHHGVRISDSALVAAAVLSDRYISGR  473
GRMZM2G162968_T01       KDPALERRFQQVYVDQPSVEDTISILRGLRERYELHHGVRISDSALVAAAVLSDRYISGR  464
                        *****************:****:***************************:*********

TaClpB1                 FLPDKAIDLVDESAAKLKMEITSKPTALDEIDRSVLKLEMERLSLTNDTDKASRDRLSRI  524
Bradi1g16190.1          FLPDKAIDLVDESAAKLKMEITSKPTALDEIDRSVLKLEMERLSLTNDTDKASKDRLSRI  519
LOC_Os03g31300.1        FLPDKAIDLVDESAAKLKMEITSKPTALDEIDRAVIKLEMERLSLTNDTDKASRDRLSRI  527
Si034086m               FLPDKAIDLVDESAAKLKMEITSKPTALDEIDRAVLKLEMERLSLTNDTDKASKGRLSRI  527
Sb01g032210.1           FLPDKAIDLVDESAAKLKMEITSKPTALDEIDRAVLKLEMERLSLTNDTDKASKDRLSRL  533
GRMZM2G162968_T01       FLPDKAIDLVDESAAKLKMEITSKPTALDEIDRAVLKIEMERLSLTNDTDKASKDRLSRL  524
                        *********************************:*:*:***************: ****:

TaClpB1                 EAELSLLKERQKGLTEQWEREKSVMTKIQSIKEEIDRLNVEIQQAEREYDLNRAAELKYG  584
Bradi1g16190.1          EAELSLLKERQNKLTEQWEHEKSVMTKIQSIKEEIDRLNVEIQQAEREYDLNRAAELKYG  579
```

```
LOC_Os03g31300.1           EAELSLLKEKQKDLTEQWEREKSVMTKIQSIKEEIDRVNVEIQQAEREYDLNRAAELKYG 587
Si034086m                  EAELSLLKDKQRELTEQWEHEKSVMTKIQSIKEEIDRVNVEIQQAEREYDLNRAAELKYG 587
Sb01g032210.1              EAELSLLKDKQRQLTERWEHEKSVMTKIQSIKEEIDRVNVEIQQAEREYDLNRAAELKYG 593
GRMZM2G162968_T01          EAELSLLKDKQRQLTEKWEHEKSVMTKIQSIKEEIDRVNVEIQQAEREYDLNRAAELKYG 584
                           ********::*. ***:**:*****************:**********************

TaClpB1                    SLNALQRDLQKTEDELNEYQSSGKSMLREEVTQDDIAEIVSRWTGIPVSKLKQSDREKLL 644
Bradi1g16190.1             SLNALQRQLQTTENELNEYQSSGKSMLREEVSQDDIAEIVSRWTGIPVSKLKQSDREKLL 639
LOC_Os03g31300.1           SLNALQRQLQTTEKELDEYQSSGKSMLREEVTQDDIAEIVSRWTGIPVSKLKQSDREKLL 647
Si034086m                  SLNALQRQLQTTEKELDEYQSSGKSMLREEVTQDDIAEIVSRWTGIPVSKLKQSDREKLL 647
Sb01g032210.1              SLNALQRQLQMTEKELDEYQSSGKSMLREEVTQDDIAEIVSRWTGIPVSKLKQSDREKLL 653
GRMZM2G162968_T01          SLNALQRQLQTTEKELDEYQSSGKSMLREEVTQDDIAEIVSRWTGIPVSKLKQSDREKLL 644
                           *******:** **.**:**************:****************************

TaClpB1                    YLEDELHKRVVGQDPAVKAVAEAIQRSRAGLSDPNRPIASFMFMGPTGVGKTELAKALAS 704
Bradi1g16190.1             YLEDELHKRVVGQDPAVKAVAEAIQRSRAGLSDPNRPIASFMFMGPTGVGKTELAKALAS 699
LOC_Os03g31300.1           YLEEELHKRVVGQDPAVKAVSEAIQRSRAGLSDPNRPIASFMFMGPTGVGKTELAKALAA 707
Si034086m                  YLEEELHKRVVGQDPAVKAVSEAIQRSRAGLSDPNRPIASFMFMGPTGVGKTELAKALAA 707
Sb01g032210.1              FLEEELHKRVVGQDPAVKAVAEAIQRSRAGLSDPNRPIASFMFMGPTGVGKTELAKALAA 713
GRMZM2G162968_T01          YLEEELHKRVVGQDPAVKAVAEAIQRSRAGLSDPNRPIASFMFMGPTGVGKTELAKALAS 704
                           :**:****************:**************************************:

TaClpB1                    FMFNTEDAVVRIDMSEYMEKHSVSRLIGAPPGYVGYEEGGQLTEAVRRRPYSVVLFDEIE 764
Bradi1g16190.1             FMFNTEDAVVRIDMSEYMEKHSVSRLIGAPPGYVGYEEGGQLTEAVRRRPYSVVLFDEIE 759
LOC_Os03g31300.1           FMFNTEEAVVRIDMSEYMEKHSVSRLIGAPPGYVGYEEGGQLTEAVRRRPYSIILFDEIE 767
Si034086m                  FMFNTEEAVVRIDMSEYMEKHSVSRLIGAPPGYVGYEEGGQLTEAVRRRPYSVVLFDEIE 767
Sb01g032210.1              FMFNTEEAVVRIDMSEYMEKHSVSRLIGAPPGYVGYEEGGQLTEAVRRRPYSVVLFDEIE 773
GRMZM2G162968_T01          FMFNTEEAVVRIDMSEYMEKHSVSRLIGAPPGYVGYEEGGQLTEAVRRRPYSVVLFDEIE 764
                           ******:*********************************************::******

TaClpB1                    KAHSDVFNVFLQILDDGRVTDSQGRKVSFTNSIIIMTSNVGSQYILNMDEEGGATDSAYE 824
Bradi1g16190.1             KAHSDVFNVFLQVLDDGRVTDSQGRKVSFTNTIIIMTSNVGSQYILNMDEVGGATDLAYE 819
LOC_Os03g31300.1           KAHGDVFNVFLQILDDGRVTDSQGRKVSFTNSIIIMTSNVGSQFILNMDEEGGSTDSAYE 827
Si034086m                  KAHSDVFNVFLQILDDGRVTDSQGRKVSFTNTIIIMTSNVGSQYILNMDEEGGSSDSAYE 827
Sb01g032210.1              KAHSDVFNVFLQILDDGRVTDSQGRKVSFTNTIIIMTSNVGSQYILNMDEEVGSSDSAYE 833
GRMZM2G162968_T01          KAHLDVFNVFLQILDDGRVTDSQGRKVSFTNSIIIMTSNVGSQYILNMDEEDGSSDSAYE 824
                           *** ********:******************:***********:******  *::* ***

TaClpB1                    SMKKRVMDAARSVFRPEFMNRVDEYIVFKPLERKQINSIVKLQLARVQKRIADRKIKLDV 884
Bradi1g16190.1             NMKKRVMDAARSVFRPEFMNRVDEYIVFKPLEREQINSIVKLQLARVQKRIADRKIKLEV 879
LOC_Os03g31300.1           NIKKRVMDAARSVFRPEFMNRIDEYIVFKPLEREQINSIVKLQLARVQKRIADRKIKLEV 887
Si034086m                  NIKKRVMDAARSVFRPEFMNRIDEYIVFKPLEREQINSIVKLQLARVQKRIADRKIKLEV 887
Sb01g032210.1              NIKKRVMDAARSVFRPEFMNRVDEYIVFKPLEREQINSIVKLQLARVQKRIADRKIKLEV 893
GRMZM2G162968_T01          NIKRRVMDAARSVFRPEFMNRVDEYIVFKPLEREQINSIVKLQLARVQKRIADRKIKLDV 884
                           .:*:*****************:***********:************************:*

TaClpB1                    SPAAIEFLGSLGYDPNYGARPVKRVLQQYVENELAKGILRGEFKDEDSISVDTQVTVPSN 944
Bradi1g16190.1             SPGAIEFLGSLGYDPNYGARPVKRVLQQYVENELAKGILRGEFKDEDSILVDTQVTVPSN 939
LOC_Os03g31300.1           SPGAVEFLGSLGYDPNYGARPVKRVIQQYVENELAKGILRGDFKDEDSILVDTQVTVPSN 947
Si034086m                  SPAAIEFLGSLGYDPNYGARPVKRVIQQYVENELAKGILRGDFKDEDSIVVDTQVTVPSN 947
Sb01g032210.1              SPGAIEFLGSLGYDPNYGARPVKRVIQQYVENELAKGILRGDFKDEDSIFVDTQVTVPSN 953
GRMZM2G162968_T01          SPGAIEFLGSLGYDPNYGARPVKRVIQQYVENELAKGILRGDFKDEDSIFVDTQVTVPSN 944
                           **.*:********************:***************:******* **********

TaClpB1                    GKLPQQKLVFRKTNEESKPAAAQDEKFLPTV      975
Bradi1g16190.1             GQLPQQNLVFRRTGEESKPAAAEDEKFLPTV      970
LOC_Os03g31300.1           GQLPQQKLVFHKMSEESAPAAAEDEKFLPAV      978
Si034086m                  GQLPQQKLVFRKVSEESK-AAAEGEKFLPAV      977
Sb01g032210.1              GQLPQQKLVFRKVGEQSK-AAAEDEKFLPAV      983
GRMZM2G162968_T01          GQLPLRKLVFQKVGEQSK-PAAEGEKFLPAV      974
                           *:** ::***::  *:*   **: *****:*
```

Supplementary Figure 1a. Multiple sequence alignment of ClpB-p (TaClpB1) protein with respective homologs in rice, maize, *Sorghum*, *Brachypodium* and *Setaria*. In the enzyme IDs, LOC_Os indicates *Oryza sativa*, Sb indicates *Sorghum bicolor*, GRMZM indicates *Zea mays*, Bradi indicates *Brachypodium distachyon* and Si indicates *Setaria italica*. The multiple sequence alignment was done using the Clustal omega program from EBI database with default parameters.
