# Supplementary material for: Differential Regulation of Genes Coding for Organelle and Cytosolic ClpATPases under Biotic and Abiotic Stresses in Wheat
Source: Front Plant Sci. 2016 Jun 28;7:929. doi: 10.3389/fpls.2016.00929 (PMC4923199; doi:10.3389/fpls.2016.00929)
Supplement: Supplementary file 3 [file Image2.PDF]

```
TaClpB3              MNPDKFTHKTNEALAAAHEMASEAGHAQLTPLHLAAALAADRSGILRQAIAHASGGNDAA  60
TaClpB2              MNPDNFTHKTNEALVAAHEAASEAGHAQITPLHLAAALAADKSGILRQAVAGASGGNASA  60
LOC_Os05g44340.1     MNPDNFTHKTNEALVAAHEIASEAGHAQLTPLHLVAALAADKGGILRQAISQASGGDAGA  60
Sb09g025900.1        MNPDNFTHKTNEALVAAHEMASEAGHAQLTPLHLAAALAADKGGILRQAITGASGGDGAA  60
GRMZM2G360681_T01    MNPDNFTHKTNEAIVGAHEIAVEAGHAQLTPLHLAAVLAADKGGILRQAITGASGGDGAA  60
                     ****:********:..*** * ******:*****.*.****:.******:: ****: .*

TaClpB3              AESFERVASAALKRLPSQSPPPDTVPASTSLVKAVRRAQSAQKSRGDSHLAVDQLLMGLL  120
TaClpB2              GDSFERVLAGALRKLPSQSPPPDSVPASTALIKAIRRAQSAQKKRGDSHLAVDQLLMGLL  120
LOC_Os05g44340.1     PDSFERVVSGALKKLPSQSPPPDSVPASTALIKVIRRAQSAQKKRGDSHLAVDQLLLGLL  120
Sb09g025900.1        GESFERVLSNALKKLPSQSPPPDSVPASTALIKVIRRAQSAQKKRGDSHLAVDQLLLGLV  120
GRMZM2G360681_T01    GDSFERVLNNSLKKLPSQSPPPDSVPASTALIKVIRRAQSAQKKRGDSHLAVDQLLLGLL  120
                      :*****   :*::*********:*****:*:*.:********.************:**:

TaClpB3              EDPQISDALKEAGISAARVKAEVEKLRGGDN-RRVESASGDTNFQALKTYGRDLVEVAGK  179
TaClpB2              EDAQIADCLKEAGVSASRVRAELDKLRGGDNSRKVESASGDTTFQALKTYGRDLVEVAGK  180
LOC_Os05g44340.1     EDSLISDCLKEAGVSAARVRAELEKLRGGEG-RKVESASGDTNFQALKTYGRDLVEQAGK  179
Sb09g025900.1        EDSQISDCLKEAGVSAARVRAELEKLRGGEG-RRVESASGDTNFQALKTYGRDLVEQAGK  179
GRMZM2G360681_T01    EDSQISDCLKEAGVSAARVRAELEKLRGGEG-RRVESASGDTNFQALKTYGRDLVEQAGK  179
                     **  *:*.*****:**:**:**::*****:  *:********.************* ***

TaClpB3              LDPVIGRDEEIRRVVRILSRRTKNNPVLIGEPGVGKTAVVEGLAQRVVRGDVPSNLLDVR  239
TaClpB2              LDPVIGRDEEIRRVVRILSRRTKNNPVLIGEPGVGKTAVVEGLAQRVVRGDVPSNLLDVR  240
LOC_Os05g44340.1     LDPVIGRDEEIRRVVRILSRRTKNNPVLIGEPGVGKTAVVEGLAQRIVRGDVPSNLLDVR  239
Sb09g025900.1        LDPVIGRDEEIRRVVRILSRRTKNNPVLIGEPGVGKTAVVEGLAQRIVRGDVPSNLLDVR  239
GRMZM2G360681_T01    LDPVIGRDEEIRRVVRILSRRTKNNPVLIGEPGVGKTAVVEGLAQRIVRGDVPSNLLDVR  239
                     **********************************************:*************

TaClpB3              LVALDMGALVAGAKYRGEFEERLKAVLKEVEEAEGKVILFIDEIHLVLGAGRTEGSMDAA  299
TaClpB2              LVALDMGALVAGAKYRGEFEERLKAVLKEVEEADGKVILFIDEIHLVLGAGRTEGSMDAA  300
LOC_Os05g44340.1     LIALDMGALVAGAKYRGEFEERLKAVLKEVEEAEGKVILFIDEIHLVLGAGRTEGSMDAA  299
Sb09g025900.1        LIALDMGALVAGAKYRGEFEERLKSVLKEVEEAEGKVILFIDEIHLVLGAGRTEGSMDAA  299
GRMZM2G360681_T01    LIALDMGALVAGAKYRGEFEERLKAVLKEVEEAEGKVILFIDEIHLVLGAGRTEGSMDAA  299
                     *:**********************:********:**************************

TaClpB3              NLFKPMLARGQLRCIGATTLEEYRKYVEKDAAFERRFQQVFVAEPSVPDTVSILRGLKEK  359
TaClpB2              NLFKPMLARGQLRCIGATTLEEYRKYVEKDAAFERRFQQVYVAEPSVADTISILRGLKEK  360
LOC_Os05g44340.1     NLFKPMLARGQLRCIGATTLEEYRKYVEKDAAFERRFQQVFVAEPSVPDTISILRGLKEK  359
Sb09g025900.1        NLFKPMLARGQLRCIGATTLEEYRKYVEKDAAFERRFQQVFVAEPSVPDTISILRGLKEK  359
GRMZM2G360681_T01    NLFKPMLARGQLRCIGATTLEEYRKYVEKDAAFERRFQQVFVAEPSVPDTVSILRGLKEK  359
                     ****************************************:****** **:*********

TaClpB3              YEGHHGVRIQDRALVIAAQLSSRYIMGRHLPDKAIDLVDEACANVRVQLDSQPEEIDNLE  419
TaClpB2              YEGHHGVRIQDRAIVVAAQLSARYIMGRHLPDKAIDLVDEACANVRVQLDSQPEEIDNLE  420
LOC_Os05g44340.1     YEGHHGVRIQDRALVVAAQLSARYIMGRHLPDKAIDLVDEACANVRVQLDSQPEEIDNLE  419
Sb09g025900.1        YEGHHGVRIQDRALVVAAQLSARYIMGRHLPDKAIDLVDEACANVRVQLDSQPEEIDNLE  419
GRMZM2G360681_T01    YEGHHGVRIQDRALVVAAQLSARYIMGRHLPDKAIDLVDEACANVRVQLDSQPEEIDNLE  419
                     *************:*:*****:**************************************

TaClpB3              RKRIQLEVELHALEKEKDKASKARLVDVRKELDDLRDKLQPLQMKYRKEKERIDEIRSLK  479
TaClpB2              RKRIQLEVELHALEKEKDKASKARLVEVRKELDDLRDKLQPLTMKYRKEKERIDEIRKLK  480
LOC_Os05g44340.1     RKRIQLEVEHHALEKEKDKASKARLVEVKKELDDLRDKLQPLTMKYRKEKERIDEIRKLK  479
Sb09g025900.1        RKRIQLEVELHALEKEKDKASKARLVEVKKELDDLRDKLQPLTMKYRKEKERIDEIRKLK  479
GRMZM2G360681_T01    RKRIQLEVELHALEKEKDKASKARLIEVRKELDDLRDKLQPLTMKYRKEKERIDEIRKLK  479
                     ********* ***************::*:************* **************.**

TaClpB3              QRREELQFTLQEAERRMDLARVADLRYGALQEVDAAIAKLEGETGENLMLTETVGPDQIA  539
TaClpB2              QRREELQFTLQEAERRMDLARVADLKYGALQEIDVAIAKLEGETGENLMLTETVGPEQIA  540
LOC_Os05g44340.1     QRREELQFTLQEAERRMDLARVADLKYGALQEIDVAIAKLESETGENLMLTETVGPEQIA  539
Sb09g025900.1        QRREELQFSLQEAERRMDLARVADLKYGALQEIDAAISKLESETGENLMLTETVGPEQIA  539
GRMZM2G360681_T01    QRREELQFTLQEAERRMDLARVADLKYGALQEIDAAISKLESETGENLMLTETVGPEQIA  539
                     ********:****************:******:*.**:***.**************:***

TaClpB3              EVVSRWTGIPVTRLGQNEKARLIGLADRLHQRVVGQYEAVNAVGEAVLRSRAGLGRPQQP  599
TaClpB2              EVVSRWTGIPVTRLGQNDKERLVGMADRLHTRVVGQTEAVNAVAEAVLRSRAGLGRPQQP  600
LOC_Os05g44340.1     EVVSRWTGIPVTRLGQNDKERLVGLADRLHQRVVGQAEAVSAVAEAVLRSRAGLGRPQQP  599
Sb09g025900.1        EVVSRWTGIPVTRLGQNDKERLVGLADRLHQRVVGQTEAVNAVAEAVLRSRAGLGRPQQP  599
GRMZM2G360681_T01    EVVSRWTGIPVTRLGQNDKERLVGLADRLHQRVVGQTEAVSAVAEAVLRSRAGLGRPQQP  599
                     *****************:* **:*:***** ***** ***.**.****************
```

```
TaClpB3               TGSFLFLGPTGVGKTELAKALAEQLFDDENLLVRIDMSEYMEQHSVARLIGAPPGYVGHE  659
TaClpB2               TGSFLFLGPTGVGKTELAKALAEQLFDDENLLVRIDMSEYMEQHSVARLIGAPPGYVGHE  660
LOC_Os05g44340.1      TGSFLFLGPTGVGKTELAKALAEQLFDDENLLVRIDMSEYMEQHSVARLIGAPPGYVGHE  659
Sb09g025900.1         TGSFLFLGPTGVGKTELAKALAEQLFDDENLLVRIDMSEYMEQHSVARLIGAPPGYVGHE  659
GRMZM2G360681_T01     TGSFLFLGPTGVGKTELAKALAEQLFDDENLLVRIDMSEYMEQHSVARLIGAPPGYVGHE  659
                      ************************************************************

TaClpB3               EGGQLTEQVRRRPYSVILFDEVEKAHVAVFNTLLQVLDDGRLTDGQGRTVDFRNTVIIMT  719
TaClpB2               EGGQLTEQVRRRPYSVILFDEVEKAHVAVFNTLLQVLDDGRLTDGQGRTVDFRNTVIIMT  720
LOC_Os05g44340.1      EGGQLTEQVRRRPYSVILFDEVEKAHVAVFNTLLQVLDDGRLTDGQGRTVDFRNTVIIMT  719
Sb09g025900.1         EGGQLTEQVRRRPYSVILFDEVEKAHVAVFNTLLQVLDDGRLTDGQGRTVDFRNTVIIMT  719
GRMZM2G360681_T01     EGGQLTEQVRRRPYSVILFDEVEKAHVAVFNTLLQVLDDGRLTDGQGRTVDFRNTVIIMT  719
                      ************************************************************

TaClpB3               SNLGAEHLLAGMVG-NSMKVARDLVMQEVRRHFRPELLNRLDEIVIFDPLSHEQLRKVAR  778
TaClpB2               SNLGAEHLLAGMVGKNSMKVARDLVMQEVRRHFRPELLNRLDEMVIFDPLSHEQLRKVAR  780
LOC_Os05g44340.1      SNLGAEHLLAGMVGKNSMKVARDLVMQEVRRHFRPELLNRLDEIVIFDPLSHEQLRKVAR  779
Sb09g025900.1         SNLGAEHLLAGMVGKNSMKVARDLVMQEVRRHFRPELLNRLDEIVIFDPLSHEQLRKVAR  779
GRMZM2G360681_T01     SNLGAEHLLAGMVGKNSMKVARDLVMQEVRRHFRPELLNRLDEIVIFDPLSHEQLRKVAR  779
                      ************** ****************************:****************

TaClpB3               LQMKDVAVRLAERGVALAVTDAALDVILSLSYDPVYGARPIRRWIEKRIVTELSKMLIRE  838
TaClpB2               LQMKDVAVRLAERGVALAVTDAALDVILSLAYDPVYGARPIRRWIEKRVVTQLSKMLIQE  840
LOC_Os05g44340.1      LQMKDVAVRLAERGVALAVTDAALDVILSLSYDPVYGARPIRRWIEKRVVTQLSKMLIQE  839
Sb09g025900.1         LQMKDVAVRLAERGIALAVTDAALDIILSLSYDPVYGARPIRRWIEKRVVTQLSKMLIQE  839
GRMZM2G360681_T01     LQMKDVAVRLAERGIALAVTDAALDIILSLSYDPVYGARPIRRWIEKRVVTQLSKMLIQE  839
                      **************:**********:****:*****************:**:******:*

TaClpB3               EIDENSTVYIDAAPSKDELTYGVDKHGGLVNARTGHKSDILIQVPSGAVGGD----AAHA  894
TaClpB2               EIDENCTVYIDAA-DKDELAYRVDRSGGLVNAETGQRSDILIQVPNGALGGGGGGEAAKA  899
LOC_Os05g44340.1      EIDENCTVYIDAAPHKDELAYRVDNRGGLVNAETGQKSDILIQVPNGAATG---SDAAQA  896
Sb09g025900.1         EIDENCTVYIDAAPAKDELAYRVDRSGGLVNAETGLKSDILIQVPNDAV-R---SDAAQA  895
GRMZM2G360681_T01     EIDENCTVYIDAAPGKDELVYRVDRSGGLVNAETGMKSDILIQVPTSST-R---SDAAQA  895
                      *****.*******  ****.* **. ******.** :********. :        **:*

TaClpB3               VKKMKIMQDSGEVDDMEEE-         913
TaClpB2               VKKMRVMEDGD-EDGMDEDA         918
LOC_Os05g44340.1      VKKMRIMEDE---DGMDEE-         912
Sb09g025900.1         VKKMRIMEEED-EDGMDEE-         913
GRMZM2G360681_T01     VKKMRIMEE-D-EDGMDEE-         912
                      ****::*::    * *:*:
```

Supplementary Figure 1b. Multiple sequence alignment of wheat ClpB-cyt (TaClpB2 and TaClpB3) proteins with their respective homologs from rice, maize, *Sorghum*, *Brachypodium* and *Setaria*. In the enzyme IDs, LOC_Os indicates *Oryza sativa*, Sb indicates *Sorghum bicolor*, GRMZM indicates *Zea mays*, Bradi indicates *Brachypodium distachyon* and Si indicates *Setaria italica*. The multiple sequence alignment was done using the Clustal omega program from EBI database with default parameters.
