# Supplementary material for: Differential Regulation of Genes Coding for Organelle and Cytosolic ClpATPases under Biotic and Abiotic Stresses in Wheat
Source: Front Plant Sci. 2016 Jun 28;7:929. doi: 10.3389/fpls.2016.00929 (PMC4923199; doi:10.3389/fpls.2016.00929)
Supplement: Supplementary file 4 [file Image3.PDF]

```
LOC_Os12g12850.1      MAGTLLQPVALGTTFAGRVSGQRWKSHGTRRPPSMLAMSLSRPVKMAAFVGLRSVHSFSV   60
GRMZM2G009443_T01     MAGTLLQSVALGTPFGGRMRTHCWRSHGTRRPASMLAVSLSRPVKMSAFVGLRSVHSFSV   60
Si021139m             MAGTLLQSVALGTTFGGRISTQRWRSHGTRRPASMLAMSLSRPVKMSAFVGLRSVHSFSV   60
Sb08g007750.1         MAGTLLQSVALGTTFGGRISTQRWRSHGARRPASMLAMSLSRPVKMSAFVGLRSVHSFSV   60
TaClpC1               MAGTLVLQVALETTFAGRTGGRHCKSHGTRRTPSMLAMSLNRPVRMAAFVGLRSVHSFSA   60
Bradi4g39880.1        MAGTLLLPVALGSTFAGRASGQRWKSHGTRRPASMLAMSLNRPVRMAAFVGLRSAHSFTA   60
                      *****:  *** : *.**   :  :***:**  ****:**.***:*:*******.***:.

LOC_Os12g12850.1      TPV-TNFRSTVASYRSR-RGRRARFVTRSMFERFTEKAIKVIMLAQEEARRLGHNFVGTE   118
GRMZM2G009443_T01     TPTVSNSRSAVASYRSSRQTRRSRFVTQAMFERFTEKAIKVIMLAQEEARRLGHNFVGTE   120
Si021139m             TPTVSNSRSAVASYRSSRRTRRSRFVTRAMFERFTEKAIKVIMLAQEEARRLGHNFVGTE   120
Sb08g007750.1         TPTVSNSRSTVASYRSSRRTRRSRFVTRAMFERFTEKAIKVIMLAQEEARRLGHNFVGTE   120
TaClpC1               VR-SSNFRSTVASYRFSRRGRRASFVTRAMFERFTEKAIKVIMLAQEEARRLGHNFVGTE   119
Bradi4g39880.1        TP-ASNYRSTVASYRSSRRGRRTRFVTRAMFERFTEKAIKVIMLAQEEARRLGHNFVGTE   119
                      .   :* **:*****   : **: ***::*******************************

LOC_Os12g12850.1      QILLGLIGEGTGIAAKVLKSMGINLKDARVEVEKIIGRGNGFVAVEIPFTPRAKRVLELS   178
GRMZM2G009443_T01     QILLGLIGEGTGIAAKVLKSMGINLKDARVEVEKIIGRGNGFVAVEIPFTPRAKRVLELS   180
Si021139m             QILLGLIGEGTGIAAKVLKSMGINLKDARVEVEKIIGRGNGFVAVEIPFTPRAKRVLELS   180
Sb08g007750.1         QILLGLIGEGTGIAAKVLKSMGINLKDARVEVEKIIGRGNGFVAVEIPFTPRAKRVLELS   180
TaClpC1               QVLLGLVGEGTGIAAKVLKSMGINLKDARVEVEKIIGRGNGFVAVEIPFTPRAKRVLELS   179
Bradi4g39880.1        QILLGLVGEGTGIAAKVLKSMGINLKDARVEVEKIIGRGNGFVAVEIPFTPRAKRVLELS   179
                      *:****:*****************************************************

LOC_Os12g12850.1      LEEARQLGHNYIGSEHLLLGLLREGEGVAARVLESLGADPSNIRTQVIRMIGETTEAVGA   238
GRMZM2G009443_T01     LEEARQLGHNYIGSEHLLLGLLREGEGVAARVLESLGADPSNIRTQVIRMIGETTEAVGA   240
Si021139m             LEEARQLGHNYIGSEHLLLGLLREGEGVAARVLESLGADPSNIRTQVIRMIGETTEAVGA   240
Sb08g007750.1         LEEARQLGHNYIGSEHLLLGLLREGEGVAARVLESLGADPSNIRTQVIRMIGETTEAVGA   240
TaClpC1               LEEARQLGHNYIGSEHLLLGLLREGEGVAARVLESLGADPSNIRTQVVRMIGETTEAVGA   239
Bradi4g39880.1        LEEARQLGHNYIGSEHLLLGLLREGEGVAARVLESLGADPSNIRTQVVRMIGETTEAVGA   239
                      ***********************************************:************

LOC_Os12g12850.1      GVGGGSSGNKMPTLEEYGTNLTKLAEEGKLDPVVGRQPQIERVVQILGRRTKNNPCLIGE   298
GRMZM2G009443_T01     GVGGGSSGNKMPTLEEYGTNLTKLAEEGKLDPVVGRQPQIERVVQILGRRTKNNPCLIGE   300
Si021139m             GVGGGSSGNKMPTLEEYGTNLTKLAEEGKLDPVVGRQPQIERVVQILGRRTKNNPCLIGE   300
Sb08g007750.1         GVGGGSSGNKMPTLEEYGTNLTKLAEEGKLDPVVGRQPQIERVVQILGRRTKNNPCLIGE   300
TaClpC1               GVGGGSSGNKMPTLEEYGTNLTKLAEEGKLDPVVGREPQIERVVQILGRRTKNNPCLIGE   299
Bradi4g39880.1        GVGGGSSGNKMPTLEEYGTNLTKLAEEGKLDPVVGRQPQIERVVQILGRRTKNNPCLIGE   299
                      ************************************:***********************

LOC_Os12g12850.1      PGVGKTAIAEGLAQRISTGDVPETIEGKKVITLDMGLLVAGTKYRGEFEERLKKLMEEIK   358
GRMZM2G009443_T01     PGVGKTAIAEGLAQRISTGDVPETIEGKKVITLDMGLLVAGTKYRGEFEERLKKLMEEIK   360
Si021139m             PGVGKTAIAEGLAQRISTGDVPETIEGKKVITLDMGLLVAGTKYRGEFEERLKKLMEEIK   360
Sb08g007750.1         PGVGKTAIAEGLAQRISTGDVPETIEGKKVITLDMGLLVAGTKYRGEFEERLKKLMEEIK   360
TaClpC1               PGVGKTAIAEGLAQRISSGDVPETIEGKKVITLDMGLLVAGTKYRGEFEERLKKLMEEIK   359
Bradi4g39880.1        PGVGKTAIAEGLAQRISSGDVPETIEGKKVITLDMGLLVAGTKYRGEFEERLKKLMEEIK   359
                      *****************:******************************************

LOC_Os12g12850.1      QSDEIILFIDEVHTLIGAGAAEGAIDANILKPALARGELQCIGATTLDEYRKHIEKDPA   418
GRMZM2G009443_T01     QSDEIILFIDEVHTLIGAGAAEGAIDANILKPALARGELQCIGATTLDEYRKHIEKDPA   420
Si021139m             QSDEIILFIDEVHTLIGAGAAEGAIDANILKPALARGELQCIGATTLDEYRKHIEKDPA   420
Sb08g007750.1         QSDEIILFIDEVHTLIGAGAAEGAIDANILKPALARGELQCIGATTLDEYRKHIEKDPA   420
TaClpC1               QSDEIILFIDEVHTLIGAGAAEGAIDANILKPALARGELQCIGATTLDEYRKHVEKDPA   419
Bradi4g39880.1        QSDEIILFIDEVHTLIGAGAAEGAIDANILKPALARGELQCIGATTLDEYRKHVEKDPA   419
                      ******************************************************:*****

LOC_Os12g12850.1      LERRFQPVRVPEPTVDETIEILRGLRERYEIHHKLRYTDDALISAAKLSYQYISDRFLPD   478
GRMZM2G009443_T01     LERRFQPVKVPEPTVDETIEILRGLRERYEIHHKLRYTDEALIAAAKLSYQYISDRFLPD   480
Si021139m             LERRFQPVKVPEPTVDETIEILRGLRERYEIHHKLRYTDEALIAAAKLSYQYISDRFLPD   480
Sb08g007750.1         LERRFQPVKVPEPTVDETIEILRGLRERYEIHHKLRYTDEALIAAAKLSYQYISDRFLPD   480
TaClpC1               LERRFQPVKVPEPSVDETIEILRGLRERYEIHHKLRYTDDSLIAAAKLSYQYISDRFLPD   479
Bradi4g39880.1        LERRFQPVKVPEPSVDETIEILRGLQERYEIHHKLRYTDDALIAAAKLSYQYISDRFLPD   479
                      ********:****:***********:*************::**:****************

LOC_Os12g12850.1      KAIDLIDEAGSRVRLRHAQVPEEARELDKELKQITKDKNEAVRSQDFEKAGELRDREMEL   538
GRMZM2G009443_T01     KAIDLIDEAGSRVRLQHAQVPEEARELDKELKQVTKQKNEAVRSQDFEKAGELRDREMEL   540
Si021139m             KAIDLIDEAGSRVRLQHAQVPEEARELDKELKQVTKQKNEAVRSQDFEKAGELRDREMEL   540
Sb08g007750.1         KAIDLIDEAGSRVRLQHAQVPEEARELDKELKQITKQKNEAVRSQDFEKAGELRDREMEL   540
TaClpC1               KAIDLIDEAGSRVRLRHAQVPEEARELDKELKQITKDKNEAVRGQDFEKAGELRDREMEL   539
Bradi4g39880.1        KAIDLIDEAGSRVRLRHAQVPEEARELDKELKQITKDKNEAVRGQDFEKAGELRDREMEL   539
                      ***************:*****************:**:*****.*****************
```

```
LOC_Os12g12850.1        KAQITALIDKSKEMSKAETESGETGPLVNEADIQHIVSSWTGIPVEKVSSDESDKLLKME    598
GRMZM2G009443_T01       KAQITALIDKSKELSKAEEESGETGPMVNEEDIQHIVSSWTGIPVEKVSSDESDKLLKME    600
Si021139m               KAQITAFIDKSKEMSKAEEESGETGPMVNEADIQHIVSSWTGIPVEKVSSDESDKLLKME    600
Sb08g007750.1           KAQITAFIDKSKELSKAEEESGETGPMVNEADIQHIVSSWTGIPVEKVSSDESDKLLKME    600
TaClpC1                 KAQITALIDKSKEMNKAETESGETGPMVHESDIQHIVSSWTGIPVEKVSTDESDKLLKME    599
Bradi4g39880.1          KAQITALIDKSKEMIKAETDSGETGPMVTEADIQHIVSSWTGIPVEKVSTDESDKLLKME    599
                        ******:******: *** :******:* * ******************:**********

LOC_Os12g12850.1        ETLHQRVIGQDEAVKAISRSIRRARVGLKNPNRPIASFIFAGPTGVGKSELAKALAAYYF    658
GRMZM2G009443_T01       ETLHKRVIGQDEAVVAISRSIRRARVGLKNPNRPIASFIFAGPTGVGKSELAKALAAYYF    660
Si021139m               ETLHKRVIGQDEAVVAISRSIRRARVGLKNPNRPIASFIFAGPTGVGKSELAKALAAYYF    660
Sb08g007750.1           ETLHTRVIGQDEAVVAISRSIRRARVGLKNPNRPIASFIFAGPTGVGKSELAKALAAYYF    660
TaClpC1                 ETLHKRVIGQDEAVKAISRSVRRARVGLKSPNRPIASFIFAGPTGVGKSELAKTLASYYF    659
Bradi4g39880.1          ETLHKRVIGQDEAVKAISRSVRRARVGLKNPNRPIASFIFAGPTGVGKSELAKALASYYF    659
                        **** ********* *****:********.***********************:**:***

LOC_Os12g12850.1        GSEEAMIRLDMSEFMERHTVSKLIGSPPGYVGYTEGGQLTEAVRRRPYTVVLFDEIEKAH    718
GRMZM2G009443_T01       GSEEAMIRLDMSEFMERHTVSKLIGSPPGYVGYTEGGQLTEAVRRRPYTVVLFDEIEKAH    720
Si021139m               GSQEAMIRLDMSEFMERHTVSKLIGSPPGYVGYTEGGQLTEAVRRRPYTVVLFDEIEKAH    720
Sb08g007750.1           GSEEAMIRLDMSEFMERHTVSKLIGSPPGYVGYTEGGQLTEAVRRRPYTVVLFDEIEKAH    720
TaClpC1                 GSEEAMIRLDMSEFMERHTVSKLIGSPPGYVGYTEGGQLTEAVRRRPYSVVLFDEIEKAH    719
Bradi4g39880.1          GSEEAMIRLDMSEFMERHTVSKLIGSPPGYVGYTEGGQLTEAVRRRPYSVVLFDEIEKAH    719
                        **:*********************************************:***********

LOC_Os12g12850.1        PDVFNMMLQILEDGRLTDSKGRTVDFKNTLLIMTSNVGSSVIEKGGRKIGFDLDYDEKDS    778
GRMZM2G009443_T01       PDVFNMMLQILEDGRLTDSKGRTVDFKNTLLIMTSNVGSSVIEKGGRKIGFDLDSDEKDS    780
Si021139m               PDVFNMMLQILEDGRLTDSKGRTVDFKNTLLIMTSNVGSSVIEKGGRKIGFDLDSDEKDS    780
Sb08g007750.1           PDVFNMMLQILEDGRLTDSKGRTVDFKNTLLIMTSNVGSSVIEKGGRKIGFDLDSDEKDS    780
TaClpC1                 PDVFNMMLQILEDGRLTDSKGRTVDFKNTLLIMTSNVGSSVIEKGGRKIGFDLDSDEKDS    779
Bradi4g39880.1          PDVFNMMLQILEDGRLTDSKGRTVDFKNTLLIMTSNVGSSVIEKGGRKIGFDLDSDEKDS    779
                        ****************************************************** *****

LOC_Os12g12850.1        SYSRIKSLVVEEMKQYFRPEFLNRLDEMIVFRQLTKLEVKEIAEIMLKEVFDRLKAKDID    838
GRMZM2G009443_T01       SYSRIKSLVIEEMKQYFRPEFLNRLDEMIVFRQLTKLEVKEIADIMLQEVFDRLKAKDIN    840
Si021139m               SYGRIKSLVIEEMKQYFRPEFLNRLDEMIVFRQLTKLEVKEIADIMLQEVFDRLKAKDIN    840
Sb08g007750.1           SYSRIKSLVIEEMKQYFRPEFLNRLDEMIVFRQLTKLEVKEIAEIMLKEVFDRLKAKDIN    840
TaClpC1                 SYGRIKSLVIEEMKQYFRPEFLNRLDEMIVFRQLTKLEVKEIADIMLQEVFTRLKTKDIN    839
Bradi4g39880.1          SYGRIKSLVVEEMKQYFRPEFLNRLDEMIVFRQLTKLEVKDIAEIMLLEVFNRLKAKEIN    839
                        **.******:******************************:**:*** *** ***:*:*:

LOC_Os12g12850.1        LQVTEKFKERIVDEGFNPSYGARPLRRAIMRLLEDSLAEKMLAGEVKEGDSAIVDVDSEG    898
GRMZM2G009443_T01       LQVTEKFKERVVDEGYNPSYGARPLRRAIMRLLEDSLAEKMLAGEVKEGDSAIVDVDSEG    900
Si021139m               LQVTEKFKERVVDEGYNPSYGARPLRRAIMRLLEDSLAEKMLAGEVKEGDSAIVDVDSEG    900
Sb08g007750.1           LQVTEKFKERVVDEGYNPSYGARPLRRAIMRLLEDSLAEKMLAGEVKEGDSAIVDVDSEG    900
TaClpC1                 LQVTEKFKERVVDEGYNPSYGARPLRRAIMRLLEDSLAEKILGGEVKEGDSVIVDVDPEG    899
Bradi4g39880.1          LQVTEKFKERVVDEGYNPSYGARPLRRAIMRLLEDSLAEKILAGEVKEGDSAIVDVDSEG    899
                        **********:****:************************:*.********.***** **

LOC_Os12g12850.1        KVIVLNGQSGLPELSTPAVTV   919
GRMZM2G009443_T01       KVVVLNGQGGIPELSTPAITV   921
Si021139m               KVIVLNSQGGIPELPTPAVTV   921
Sb08g007750.1           KVVVLNGQGGIPELSTPAITV   921
TaClpC1                 KVIVLNGESGLPELPTPAVAV   920
Bradi4g39880.1          KVIVLNGESGLPELPTPAVTV   920
                        **:***.:.*:*** ***::*
```

Supplementary Figure 1c Multiple sequence alignment of TaClpC$_1$ protein with their respective homologs in rice, maize, *Sorghum*, *Brachypodium* and *Setaria*. In the enzyme IDs, LOC_Os indicates *Oryza sativa*, Sb indicates *Sorghum bicolor*, GRMZM indicates *Zea mays*, Bradi indicates *Brachypodium distachyon* and Si indicates *Setaria italica*. The multiple sequence alignment was done using the Clustal omega program from EBI database with default parameters.
